# Network Pharmacology-Guided Development of a Novel Integrative Regimen to Prevent Acute Graft-vs.-Host Disease

**DOI:** 10.3389/fphar.2018.01440

**Published:** 2018-12-13

**Authors:** Ming Lyu, Zhengcan Zhou, Xiaoming Wang, Hong Lv, Mei Wang, Guixiang Pan, Yuefei Wang, Guanwei Fan, Xiumei Gao, Yuxin Feng, Yan Zhu

**Affiliations:** ^1^Tianjin State Key Laboratory of Modern Chinese Medicine, Tianjin University of Traditional Chinese Medicine, Tianjin, China; ^2^Research and Development Center of TCM, Tianjin International Joint Academy of Biotechnology & Medicine, TEDA, Tianjin, China; ^3^State Key Laboratory of Experimental Hematology, Institute of Hematology & Blood Diseases Hospital, Chinese Academy of Medical Science & Peking Union Medical College, Tianjin, China; ^4^Medical Experiment Center, First Teaching Hospital of Tianjin University of Traditional Chinese Medicine, Tianjin, China

**Keywords:** acute graft vs. host disease, network pharmacology, Xuebijing injection, cyclosporin A, integrative medicine

## Abstract

Lapses in the graft-vs.-host disease (GVHD) prophylaxis and side effects of current standard care following allogeneic hematopoietic stem cell transplantation (allo-HSCT) call for novel regimens. Traditional approaches targeting T cells showed limited success in preventing acute GVHD (aGVHD). System medicine showed promising results treating complex diseases such as sepsis and multi-organ dysfunction syndrome (MODS). Adapting established network pharmacology analysis methods, we aimed to develop novel integrative regimens to prevent aGVHD. Our network pharmacology analysis predicted that Xuebijing injection (XBJ) targets a series of key node proteins in aGVHD network. It also unveiled that *Salviae miltiorrhizae* (Danshen), an herb in Xuebijing formula, which prevented aGVHD in rats, shares five out of six key GVHD node proteins targeted by XBJ. Interestingly, network pharmacology analysis indicated Xuebijing may share multiple aGVHD targets with Cyclosporin A (CsA), a first-line drug for preventing aGVHD in the clinic. Based on current information, we hypothesized that combination of XBJ and CsA may yield superior results in aGVHD prevention than either drug alone. We performed *in vitro* and *in vivo* assays to validate the predictions by the network pharmacology analysis. *In vitro* assays revealed XBJ prevented platelet aggregation and NF-κB nuclear translocation in macrophages. XBJ also promoted angiogenesis in tube-formation assay. Importantly, the combination of CsA and XBJ was effective in rescuing mice subjected to lethal GVHD. XBJ contributed to the rescue through preventing NF-κB nuclear translocation, attenuating inflammation and maintaining viability of macrophages. Overall, network pharmacology is a powerful tool to develop novel integrative regimens. Combination of XBJ and CsA may shed light on preventing aGVHD.

## Highlights

- Network pharmacology-based approach was used to predict and design novel regimens of integrative medicine for graft-vs. -host disease (GVHD) prophylaxis.
- The combination of Cyclosporin A and Xuebijing injection is safe and effective in rescuing mice subjected to lethal GVHD and likely to vendor more benefits to patients than either drug alone.
- Systemic intervention utilizing integrative medicine is promising to prevent GVHD.

## Introduction

Graft-vs.-host disease (GVHD) is the leading cause of procedure related mortality and morbidity after allo-HSCT (Al-Homsi et al., 2015, 2017b). Current GVHD prevention strategies that suppress or deplete T cells are partially effective, posing side effects and revoking graft-vs. tumor (GvT) effect (Remberger et al., 2011; Holtan et al., 2014).

Immunosuppressive drugs represented by CsA are effective to a varying degree in aGVHD prevention, but often cause serious side effects such as renal and hepatic dysfunction and hypertension in patients (Atkinson et al., 1983; Hows et al., 1983; Storb et al., 1985). CsA also increases the relapse rate in cancer and the incidence of marrow engraftment failure (Atkinson et al., 1983; Penn, 1987). Only one- to two-thirds of treated patients responded to current prevention regimens for aGVHD, calling for novel strategies producing better effectiveness with fewer treatment-related side effects.

Emerging alternative strategies to prevent GVHD include post-transplantation cyclophosphamide (PTC; Luznik et al., 2001; Luznik and Fuchs, 2010), co-stimulation blocking agents (Blazar et al., 1994), chemo-cytokine antagonists (Reshef et al., 2012), proteasome inhibitors (Al-Homsi et al., 2016, 2017a,b), and epigenetic modulators (Reddy et al., 2008; Choi et al., 2010a). Some of these approaches may maintain GvT effect or comprise cytotoxic activity to prevent disease relapse.

Network pharmacology is becoming a powerful tool to study complex diseases and Chinese medicine, revealing the complex relationships among proteins, drugs, and disease phenotypes (Hopkins, 2007; Leung et al., 2013; Li and Zhang, 2013; Li, 2016). Molecular connectivity maps have been established to link comprehensive knowledge between molecules of interest in a given biological context. This is particularly valuable to study Chinese medicine, empowering researchers to clarify their visions on candidate compounds that lead to the identification of major active compounds and potential applications in a complex network (Liu et al., 2016; Suo et al., 2017). Combining a system-level pharmacological investigation with experimental validations facilitates discoveries of the potential active ingredients and action mechanisms of Traditional Chinese Medicine (TCM), formulating novel paradigms for the application of Chinese medicine (Lyu et al., 2017).

Xuebijing injection (XBJ), a Chinese medicine formula combining 5 herbs, is routinely used as an add-on to conventional therapy to treat sepsis and septic shock in China (Jiang et al., 2013; Shi et al., 2017; Li et al., 2018). Combination of XBJ and the standard treatment yielded better clinical outcomes than the standard treatment alone (Shao et al., 2011; Gao et al., 2015). It is believed that XBJ exerts its effects on a variety of targets at different stages of a systematic infection. For example, it reduces serum TNF-α and IL-6 concentrations in patients and in preclinical models of sepsis (Fang and Wang, 2013; Jiang et al., 2013; Chen et al., 2018b), antagonizes LPS, regulates CD4+ T cell differentiation (Chen et al., 2018b), and prevents/controls the disseminated intravascular coagulation (DIC) and infections. Benefiting patients of sepsis, these effects may improve the life of patients receiving allo-HSCT in theory.

This work tested the application of network pharmacology in developing novel regimens to prevent aGVHD and confirmed our predicted candidate, Xuebijing, can rescue lethal aGVHD in a preclinical model. To our knowledge, this is the first report that a China Food and Drug Administration (CFDA) approved Chinese medicine injection is beneficial to aGVHD.

## Materials and Methods

### Chemicals and Reagents

Xuebijing injection (catalog number: z20039833, batch number: 1606121) was supplied by Tianjin Chase Sun Pharmaceutical Co., LTD (Tianjin, China). This Chinese medicine is approved by China Food and Drug Administration (CFDA) for treating sepsis and septic shock with CFDA ratification number of GuoYaoZhunZi-Z20039833 for market approval as a drug product. It is routinely used as an add-on to the conventional therapy to treat sepsis and septic shock in China (Jiang et al., 2013; Chen et al., 2018b). This injection contains extracts of 5 herbs, including *Carthami Flos* (the corolla of *Carthamus tinctorius L*.), *Paeoniae Radix Rubra* (the root of *Paeonia veitchiiLynch*.), *Chuanxiong Rhizoma* (the root of *Ligusticum chuanxiong Hort*.), *Salviae miltiorrhizae* (the root of *Salvia miltiorrhiza Bge*.), and *Angelicae sinensis Radix* [the root of *Angelica sinensis (Oliv.) Diels*.]. Methods of extraction, preparation, and quality control of XBJ were the same as reported previously (Huang et al., 2011; Cheng et al., 2016; Li et al., 2016b). Briefly, ingredients from *Carthami Flos* (“Honghua” in Chinese) were first extracted with ethanol then with water. Ingredients from the other four herbs were extracted with water. Finally, XBJ was standardized to contain 1.0–1.7 mg/mL of paeoniflorin and 0.2–0.5 mg/mL of hydroxysafflor yellow A as described (Huang et al., 2011; Cheng et al., 2016; Li et al., 2016b). All chemicals used in the experiments were ordered from Sigma-Aldrich (St. Louis, MI, USA) unless specifically indicated. The FITC labeled anti-mouse H-2Kb and PE labeled anti-mouse H-2Kd antibodies were ordered from BioLegend (San Diego, CA, USA).

### Experimental Animals

This study was carried out in accordance with the recommendations of the Guide for the Care and Use of Laboratory Animals (NIH Publication No. 85-23, revised 1996, USA) and the guidelines of Tianjin University of Traditional Chinese Medicine Animal Research Committee. The protocol was approved by the Tianjin University of Traditional Chinese Medicine Animal Research Committee (TCM-LAE-20170016).

All transplantation experiments were performed with weight- (19–22 g) and sex- (female) matched 10 week-old BALB/c and C57/Blk6 mice purchased from Vital River Company (Beijing, China).

Male Sprague-Dawley rats (200~220 g, Vital River Company, Beijing, China) were used for platelet aggregation experiments. Mice and Rats were acclimated to the standard germ-free housing room under an ambient temperature of 23 ± 2°C and 40–60% relative humidity, with a diurnal cycle of 12 h light and 12 h dark at the animal facility of Tianjin International Joint Academy of Biotechnology & Medicine for 1 week before experiments. They were provided with a normal diet and water daily for the duration of experiments.

### Database Construction And Network Analysis

Acute GVHD related targets were mainly integrated from GeneCards (Stelzer et al., 2011) and Ingenuity Pathway Analysis (IPA, http://www.ingenuity.com) database (Kramer et al., 2014). Repetitive genes were automatically identified and removed by IPA software. In addition, ingredients derived from XBJ were collected from literature mining (Huang et al., 2011; Jiang et al., 2013; Guo et al., 2014; Han et al., 2017; Zuo et al., 2017a,b) and several TCM databases, such as TCMID (Xue et al., 2013) and TCMSP (Ru et al., 2014). Compounds had more than 10 targets in mammalians were selected for further analysis. The chemical name of each compound was transferred into PubChem CID or CAS number which could be recognized by IPA software. Furthermore, corresponding targets of XBJ ingredients which were not recorded by IPA database were supplemented by literatures from PubChem and databases of TCMID and TCMSP. In total, three datasets including XBJ ingredients, acute GVHD associated targets, and corresponding targets of XBJ's major ingredients, were constructed and then uploaded into the IPA system to visualize the discovery. Acute GVHD related targets and the relationship between XBJ ingredients and their corresponding targets collected in IPA database were discovered by “Build-Path Explorer” module. Upstream regulators analyses were performed to elucidate the causal inference of upstream biological causes and probable downstream effects on cellular and organismal biology (Kramer et al., 2014). “Path designer” module was used to demonstrate the network. All these network analysis steps were also appropriate for Danshen (Han et al., 2017), one of the five herbs in XBJ.

### Acute GVHD Model and Bone Marrow Transplantation

A murine acute GVHD model was recapitulated following the established protocol and bone marrow transplantations were performed as described (Cheng et al., 2013; Al-Homsi et al., 2017a,b). Briefly, bone marrow (BM) cells were gently released from the femurs and tibias of donor C57BL/6 mice and suspended in phosphate buffer saline (PBS; Fisher Scientific, Waltham, MA). Cell suspensions were then filtered through a 70-μm filter and washed with PBS to obtain particulate free, single-cell suspensions. GVHD inocula were obtained by gently crushing the spleens of either C57BL/6 mice. Splenocytes were then filtered using a 70-μm filter and washed with PBS. Cell counts were performed on hemocytometers. Recipient BALB/c mice were subjected to total body irradiation the day before transplant (Day-1). Mice received 8.5 Grays (2 fractions, 3 h apart) via a Rad Source RS-2000 irradiator (San Diego, USA). Irradiated mice received donor BM (5 × 10^6^ cells) with splenocytes (5 × 10^6^) by tail-vein injections on day 0. Mice were treated with 0.9% NaCl, or received CsA (5 mg/kg, intraperitoneally) alone, XBJ (0.2 ml/kg, subcutaneously) alone, or both at the indicated time points. Mice were monitored for weight and scored for GVHD 3 times weekly. GVHD scoring was based on weight loss, posture, activity, fur texture, skin integrity, and diarrhea and gut injury (severity score 0 to 2 for each variable, maximum index 12). Animals were euthanized if they lost >35% of their initial weight or reached a score ≥7. The experiments were terminated on Day 30. The chimerism was determined 14 days after the transplantation according to the established methods. The mono-nuclear cells were detected with a flow cytometer (BD FACS Aria II, BD Biosciences, St. Jose, CA USA) according to established methods (Cheng et al., 2013; Chen et al., 2018b).

### Ethics Statement

The institutional animal ethics committee approved this study design. Given the severity of our study, we diligently observed all mice to minimize suffering within the frames of the experimental design. All mice in the study were housed in the pathogen-free animal facility and the overall health status was checked by trained professionals at least two times per day whenever an animal's condition deteriorated (defined by, among other parameters, decreased activity, progressing hypothermia, rapid weight loss). In detail, mice were euthanized upon signs of impending decease (i.e., inability to maintain upright position/ataxia/tremor and prolonged/deep hypothermia and/or agonal breathing) by cervical dislocation.

### Endothelial Cell Vessel Formation on Matrigel Matrix

Endothelial cell vessel formation was carried out as described (Porcu et al., 2016). EA.hy926 human endothelial cells were seeded on Matrigel (BD Biosciences, San Jose, CA, USA). Matrigel was defrosted at 4°C overnight, added to each well (45 μL) of a 96-well plate, and allowed to polymerize for 30 min at 37°C. Then cells (1.8 × 10^4^/well) were seeded and incubated overnight until capillary tubes were formed. VEGF (50 ng/mL) was used as positive control in experiments. In other tests, different dilutions of XBJ were added to cells and incubated for additional 12 h. Then Calcein AM (1 μmol/L) was used to dye cytoplasm and was incubated 30 min at 37°C. Cells were photographed with HCS and pictures were saved as GIF files.

### Platelet Aggregation Experiment

Platelets were taken from male Sprague-Dawley rats (200~220 g, Vital River Company, Beijing, China). Whole blood obtained by arteriopuncture at the abdominal aorta of the rat was collected in a tube (about 10 ml) with 10% volume of ACD solution, from which the platelet-rich plasma (PRP) was isolated by centrifugation at 200 × g for 10 min at room temperature. The PRP fraction was further processed by 10 min centrifugation at 800 × g and the final isolated platelets are suspended in TBS. Specifically, platelets of 100 μL (about 10^7^ platelets) were added in each well of a 96-well plate, followed by adding 50 μL solution of the XBJ or its dilution or 50 μL 0.9% NaCl (for the negative sample), such that each well contains 150 μL liquid. The plate is then incubated in a multi-mode microplate reader (FlexStation 3, Molecular Devices LLC. Sunnyvale, CA, USA) for 10 min at 37°C in order to get a well-blended platelet-chemical mixture. Subsequently, 50 μL agonist solution was added to each well and the optical density (OD) value under 405 nm was measured continuously for 30 min at 37°C. Both types of agonist solution used in our experiments, i.e., a solution of 25 μM ADP (Sigma-Aldrich, Shanghai, China) and a solution of 0.1U thrombin (Solarbio Life Sciences, Beijing, China) containing 2.0 mM CaCl_2_. The aggregation rate (AR) at time t is derived from the OD values, i.e., AR (t) = 100% × (OD_t = 0_-OD_t_)/OD_t=0._

### NF-κB Nuclear Translocation Assay

RAW264.7 cells were seeded in a 96-well plate (1 × 10^4^/well) and cultured for 12–24 h. LPS (Sigma-Aldrich, Shanghai, China. Cat#: L4391) (100 ng/mL) were added into each well of a 96-well plate and incubated for 30 min at 37°C. Later, cells were treated with DEX or XBJ at 37°C for 12 h. One hundred microliters of 4% paraformaldehyde was added in each well of a 96-well plate; the 96-well plate was incubated for 30 min at 37°C, after washing with PBS, cells were treated with 100 μL 0.5% Triton X-100 for 30 min. Later the cells were incubated in 5% FBS for 2 h, 50 μL of primary NF-κB p65 antibody (C-20) (Abcam, Cambridge, MA, Cat #: Ab150131) diluted for 100 times with PBS containing 0.01% Tween-20 and 0.2%FBS was added into each well, then was incubated overnight at 4°C. The next morning, NF-κB p65 antibody was removed, then 50 μL of secondary antibody (Donkey Anti-Goat IgG H&L) which was diluted 300 times with PBS containing 0.01% Tween-20 and 0.2% FBS and 50 μL of Hoechst (1:10,000) (Sigma-Aldrich, Shanghai, China. Cat#:14533) were added into each well and incubated for 2 h at room temperature. After the secondary antibody was removed from each well, cells were washed with PBS. An Operetta high content imaging system (PerkinElmer Inc., Fremont, CA, USA) was used to detect fluorescent signal.

### Cell Viability

The viability of RAW264.7 cells was measured using CCK-8 kit as described (Fan et al., 2016). 8 × 10^3^ of RAW264.7 cells were seeded in each well of a 96-well plate to allow the cells to adhere to the dish for 24 h before changing medium to no serum medium and continue the culture for another 24 h. Later, different dilutions of XBJ were used to treat the synchronized cells for 24 h. Then, the viability of RAW264.7 cells was measured according to the manufacturer's protocol on a FlexStation 3 microplate reader (Molecular Devices, Sunnyvale, CA, USA).

### Statistical Analysis

The Log-rank test was used to determine the statistical significance of Kaplan-Meier survival curves. Other results were analyzed by *t-* or ANOVA tests as appropriate, using InStat version 3.06 software for Windows (GraphPad, San Diego, CA, USA). The following terminology was used to show statistical significance: ^*^P < 0.05; ^**^P < 0.01; and ^***^P < 0.001.

## Results

### Network Pharmacology Analysis Revealed XBJ as a Candidate for aGVHD Prophylaxis

**Figure 1:**
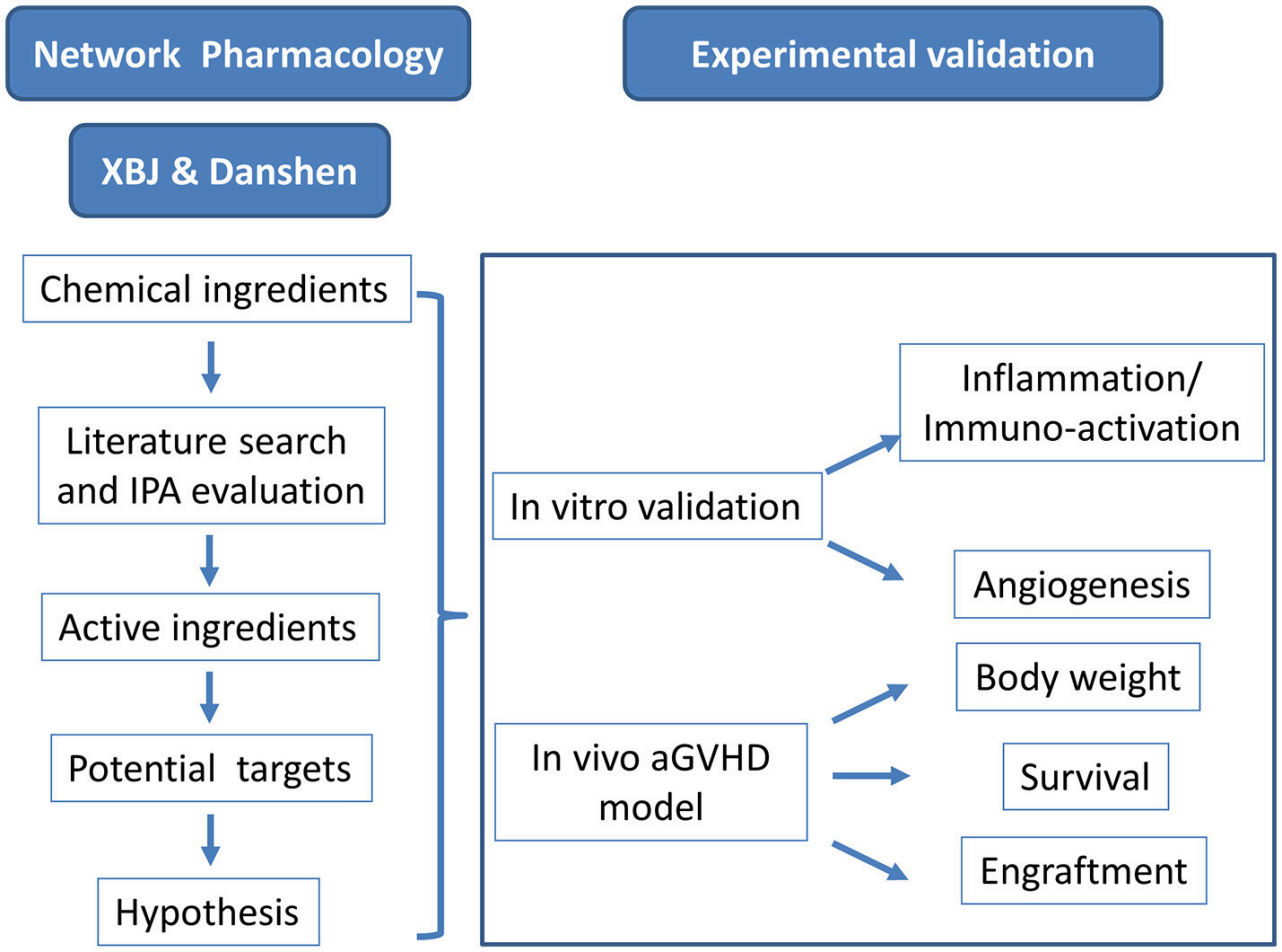
Research scheme to identify the potential application of XBJ in preventing aGVHD.

A query for XBJ targets in sepsis revealed key GVHD regulators in IPA platform (data not shown). This incident triggered a comprehensive literature mining and network pharmacology analysis to profile the potential role of XBJ and its key ingredient Danshen in aGVHD prophylaxis (Figure 1). A series of GVHD regulators emerged from this analysis as proved and potential XBJ targets.

### Network Pharmacology Analysis Predicted That Key Regulators of Acute GVHD Are Targets of XBJ

**Figure 2:**
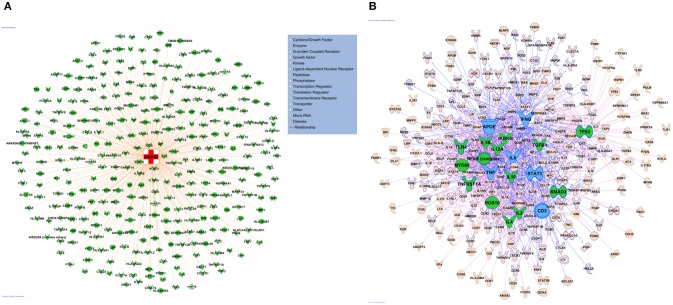
Network pharmacology analysis to identify key upstream regulators in aGVHD. **(A)** aGVHD related molecules. A total of 400 genes were collected by integrating GVHD related genes in GeneCards and IPA databases. **(B)** Key regulators in aGVHD. Top 20 upstream regulators are marked in green, the top 6 upstream regulators including TNF, IL-6, IFN-γ, APOE, STAT3, CD3 are highlighted in blue.

**Figure 3:**
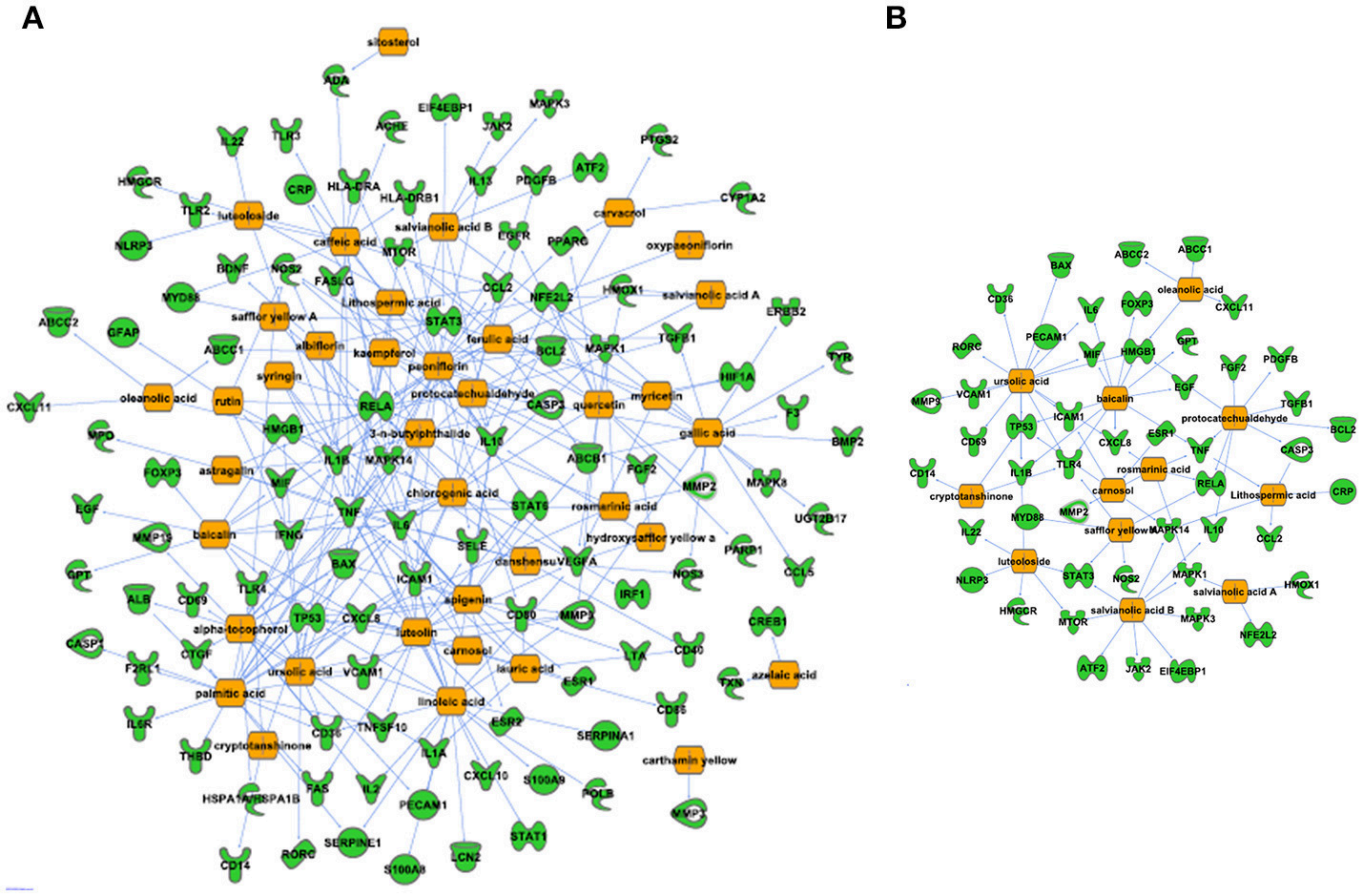
Predicted XBJ ingredient-target and Danshen ingredient-target network in aGVHD. **(A)** Predicted XBJ ingredient-target network. Thirty eight ingredients from XBJ were predicted to cooperatively modulate 110 aGVHD-related targets. **(B)** Predicted Danshen ingredient-targets network. Twelve ingredients uniquely derived from Danshen were predicted to cooperatively modulate 49 aGVHD-related targets.

**Figure 4:**
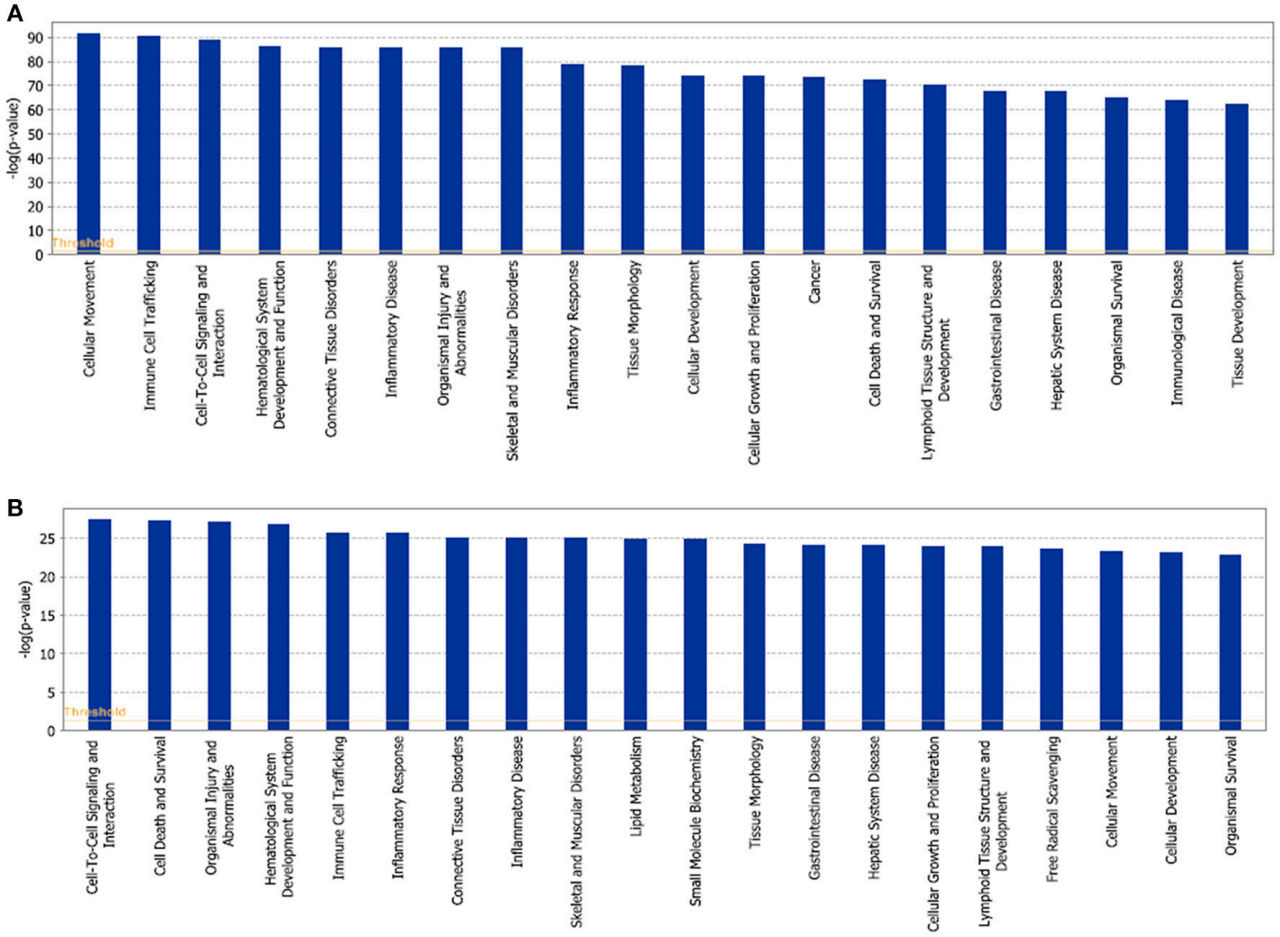
Function and related diseases of XBJ and Danshen targets. **(A)** Top 20 functions and related diseases of XBJ targets in aGVHD. **(B)** Top 20 functions and related diseases of Danshen targets in aGVHD.

**Figure 5:**
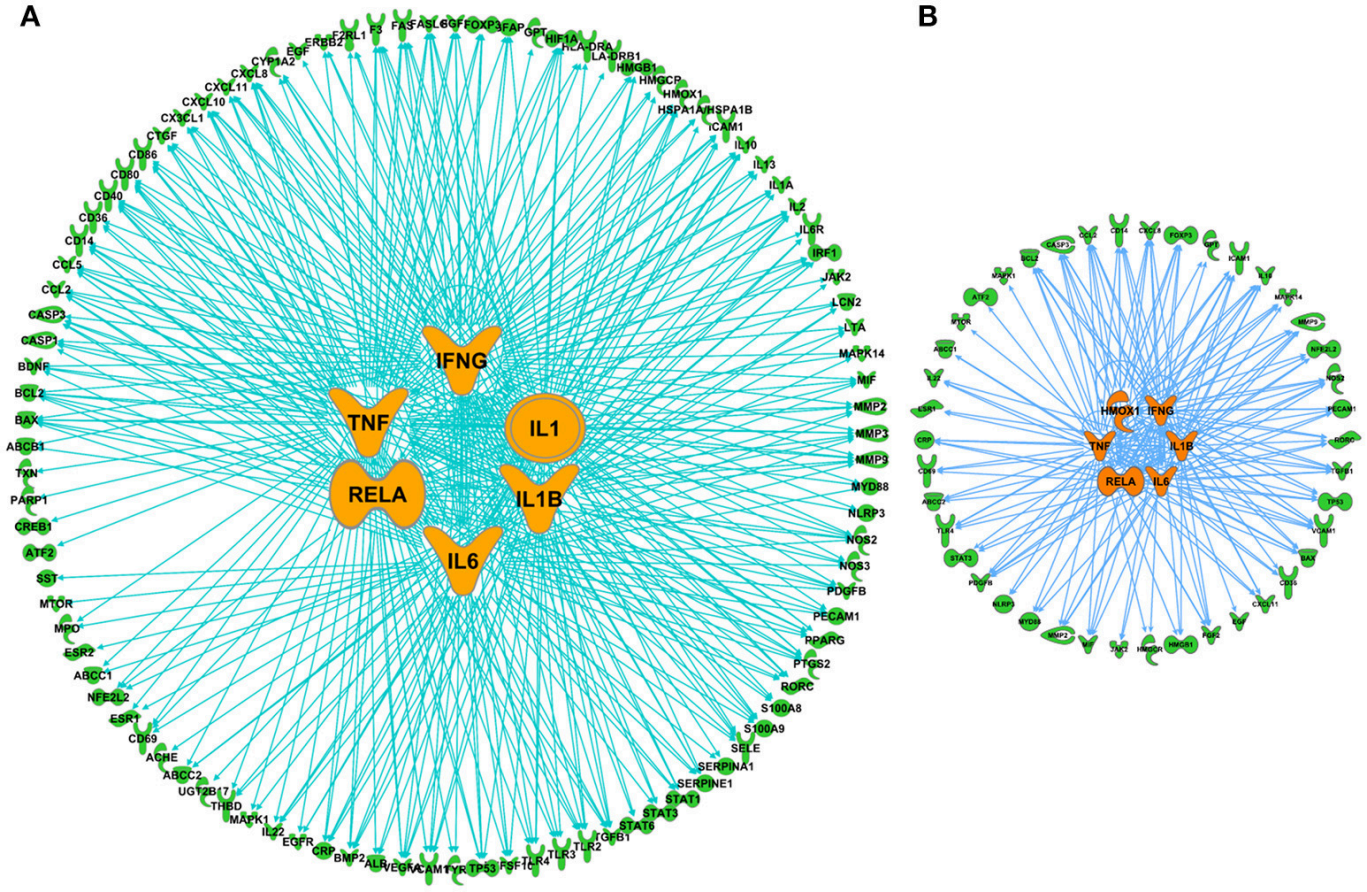
Comparison of top upstream modulators of predicted XBJ and Danshen targets in aGVHD. **(A)** The network relationship of predicted XBJ targets in aGVHD and their top six upstream modulators. The six top upstream regulators are TNF, IL-6, IFN-γ, IL-1, RELA, and IL-1B. **(B)** The network relationship of predicted Danshen targets in aGVHD and their top six upstream modulators (HMOX1, IFN-γ, TNF, RELA, IL-1β, and IL-6).

The network of 400 genes highly related to acute GVHD was constructed by the database of GeneCards and IPA (Figure 2A and Supplementary Table 1). IPA analysis revealed top 20 key upstream regulators of aGVHD. Most of them were correlated with inflammation and immunology. TNFα, IL-6, IFN-γ, and IL-1β emerged as top regulators of aGVHD predicted by IPA (Figure 2B). We found that the 38 compounds in XBJ ingredients regulate 110 aGVHD-related targets and 12 Danshen ingredients regulated 49 aGVHD-related targets. The 38 XBJ ingredients-110 aGVHD-related targets network and the 12 Danshen ingredients-49 aGVHD-related targets were established (Figure 3 and Supplementary Table 2). Comparing the function and related diseases of XBJ targets with the function and related diseases of Danshen targets, we found XBJ and Danshen targets shared 16 out of top 20 functions and related diseases (Figure 4). Furthermore, IPA analysis predicted TNFα, IL-6 and IFN-γ are among the top six key upstream regulators of the 110 XBJ targets in aGVHD (Figure 5A). Danshen shared five out of six key upstream regulators of its targets with XBJ, such as TNF, IL-6 and IFN-γ (Figure 5B). Interestingly, CsA was predicted as one of the top 6 chemical drugs that regulate XBJ targets in aGVHD, while it is not a top drug for targets of Danshen ingredients (Supplementary Figure 1). These results indicate that XBJ may target top upstream regulators in aGVHD to attenuate inflammation and cytokine storm. It may complement and enhance the function of CsA in preventing aGVHD.

### XBJ Inhibited Inflammation

**Figure 6:**
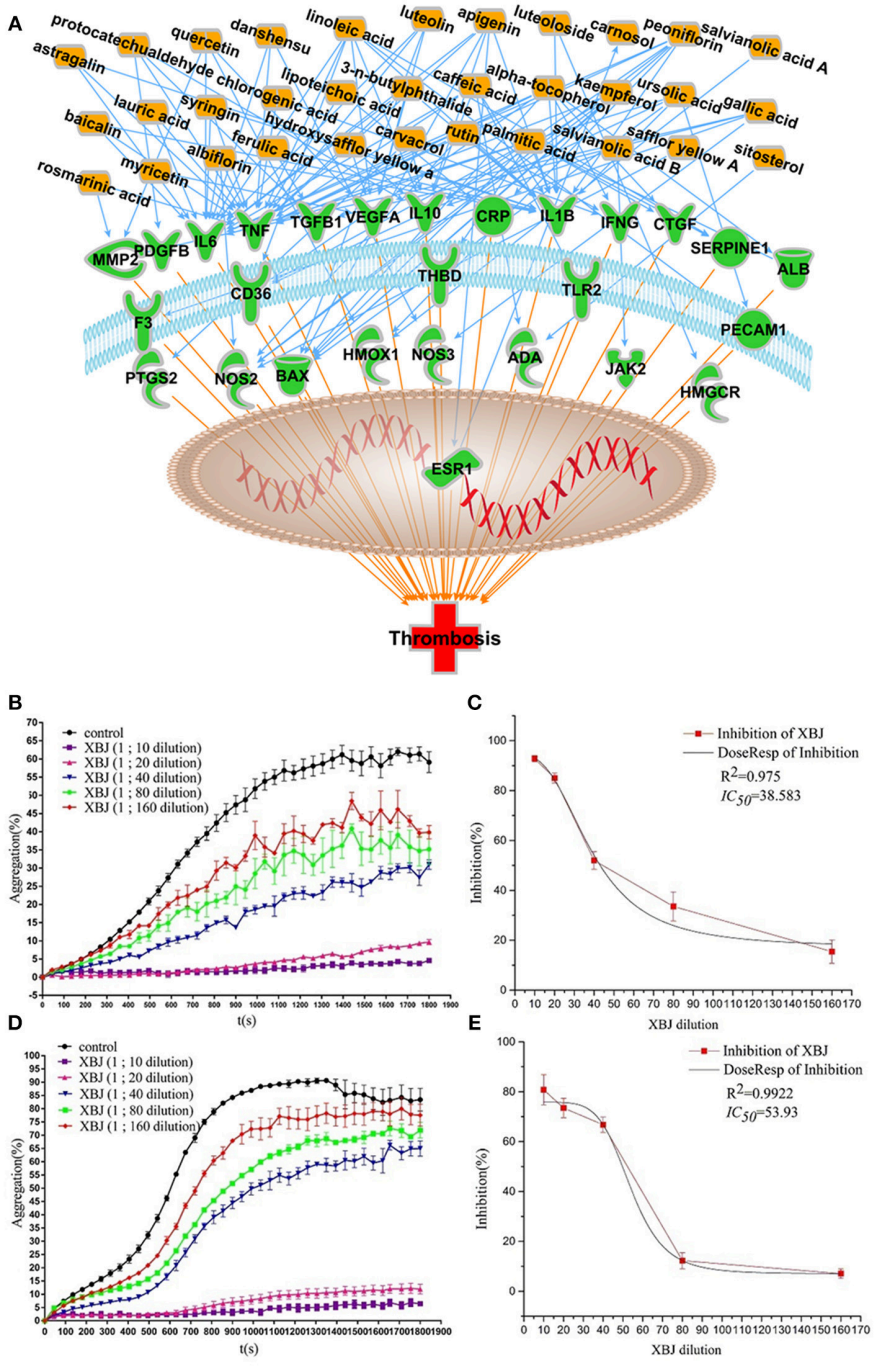
XBJ inhibits platelet aggregation. **(A)** Network pharmacology analysis of XBJ ingredients and their cellular targets in thrombosis (**B** and **C**) XBJ inhibits ADP-induced platelet aggregation. **(B)** After pre-treatment with XBJ at different dilution ratio (1/160, 1/80, 1/40, 1/20, and 1/10) for 10 min, ADP (25 μM)-induced platelet aggregation was significantly inhibited in a dose-dependently manner. **(C)** IC50 stimulation curve of XBJ (ADP) (**D** and **E**) XBJ inhibits thrombin (0.1 U)-induced platelet aggregation. **(D)** After pre-treatment with XBJ at different dilution ratio (1/160, 1/80, 1/40, 1/20, and 1/10) for 10 min, thrombin-induced platelet aggregation was significantly inhibited in a dose-dependently manner. **(E)** IC50 stimulation curve of XBJ (thrombin). The unit of IC50 is dilution ration (*P < 0.05; **P < 0.01; ***P < 0.001).

**Figure 7:**
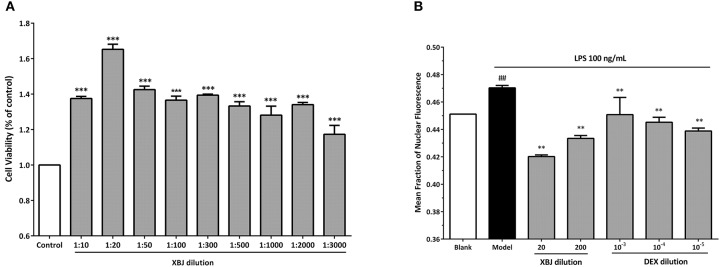
XBJ inhibits NF-κB signaling in mouse macrophages without compromising viability. **(A)** The viability of 264.7 cells treated with different doses of XBJ using CCK8 assay. XBJ was diluted at 1/10, 1/20, 1/50, 1/100, 1/300, 1/500, 1/1,000, 1/2,000, and 3,000 ratios in the experiment. *: XBJ treated groups vs. control (*P < 0.05; **P < 0.01; ***P < 0.001). **(B)** Quantification of NF-κB nuclear translocation by an Operetta high-content florescent imaging system (Perkin Elmer) after LPS stimulation in the presence of different doses of XBJ or DEX. XBJ was diluted in 1/20 and 1/200. Dexamethasone (DEX) was used as a positive control in the experiment. 10^−3^, 10^−4^, and 10^−5^mM of DEX were used to treat 264.7 cells. Cells were treated with LPS for 30 min before the indicated doses of XBJ or DEX were added. Then, the cells were incubated for 12 h before the fixation, antibody staining and imaging. ^#^: Blank vs. model. *: Treatment groups vs. model (*P < 0.05; **P < 0.01; ***P < 0.001; ^#^*P* < 0.05; ^##^*P* < 0.01; ^###^*P* < 0.001).

Our network pharmacology predictions were partially proved in our recent study (Chen et al., 2018b) and publications from other groups (Jiang et al., 2013). XBJ normalized TNFα and IL-6 production in rodent sepsis models. IPA analysis predicted 33 XBJ compounds regulate thrombosis through targeting 27 thrombosis-related factors (Figure 6A). *In vitro* assays confirmed that XBJ dose-dependently inhibits platelet aggregation. Two different experiments confirmed that XBJ dose-dependently attenuated platelet aggregation which may trigger inflammation (Figures 6B–E). NF-κB is a major downstream effector of key pro-inflammatory cytokines in aGVHD such as TNF, IL-6, and IL-1β. XBJ attenuated NF-κB nuclear translocation in immunofluorescent assays (Figure 7B and Supplementary Figure 3) without compromising the survival of mouse macrophages (Figure 7A), indicating XBJ may attenuate inflammation through NF-κB pathway.

### XBJ Promoted Angiogenesis

**Figure 8:**
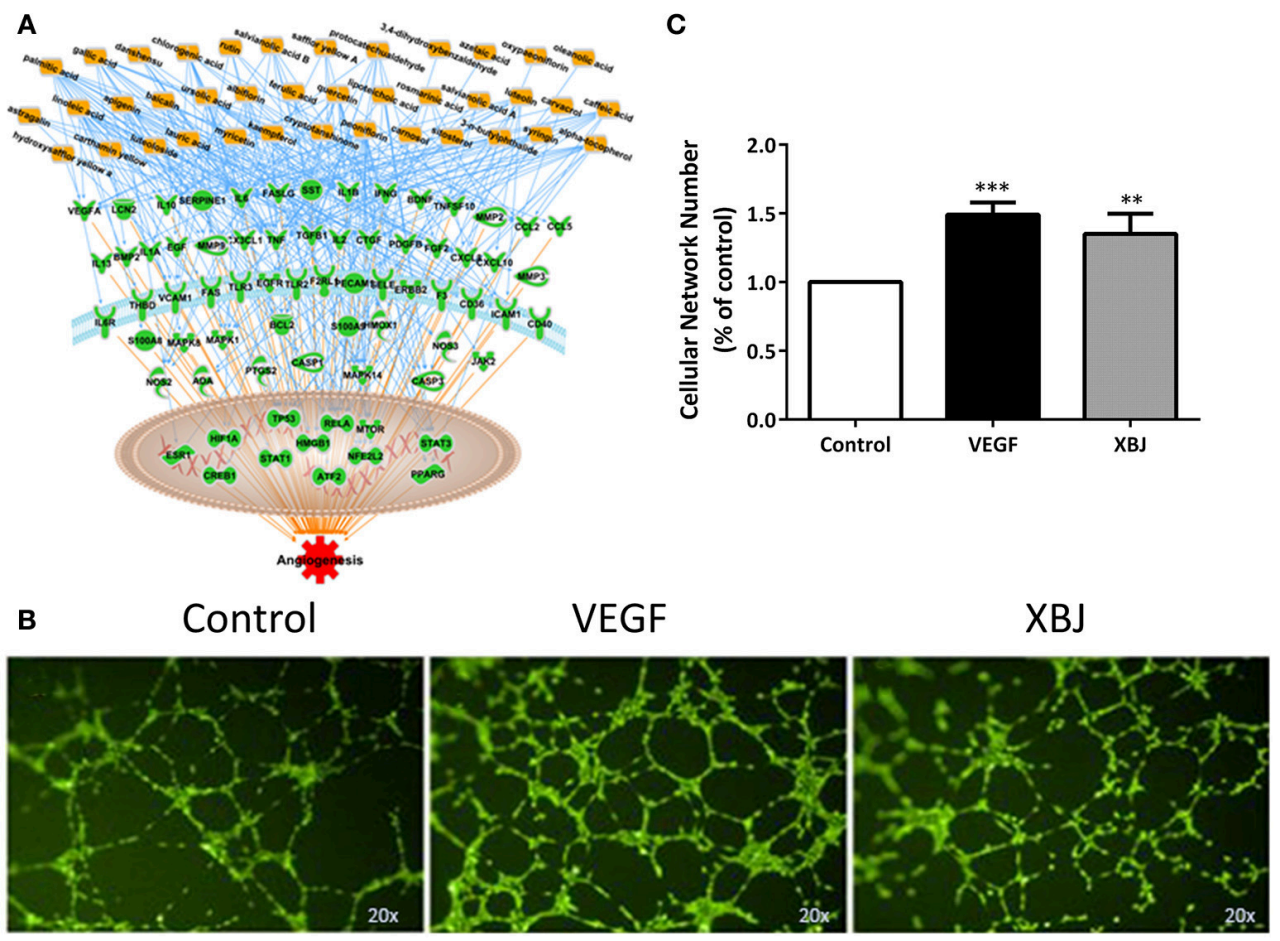
XBJ promotes angiogenesis. **(A)** Network pharmacology analysis of XBJ ingredients and their cellular targets in angiogenesis. **(B)** Representative results of tube formation assay conducted with EA.Hy926 human endothelial cells. From left to right: Control cells treated with vehicle; VEGF treated cells; XBJ (1:1,000) treated cells. The experiment was repeated for 3 times. **(C)** The quantification of tube formation assays (*P < 0.05; **P < 0.01; ***P < 0.001).

Transplant conditioning, which causes vessel damage, is another factor that worsens aGVHD and compromises hematopoietic stem cell engraftment. Network pharmacology analysis revealed multiple compounds in XBJ ingredients that may also regulate angiogenesis (Figure 8A). Consistently, XBJ promoted tube-formation *in vitro*, indicating it may promote post-injury vascular regeneration after the transplant (Figure 8).

### Combination of XBJ and CsA Prevented Acute GVHD and Improved Survival in a Murine Model

**Figure 9:**
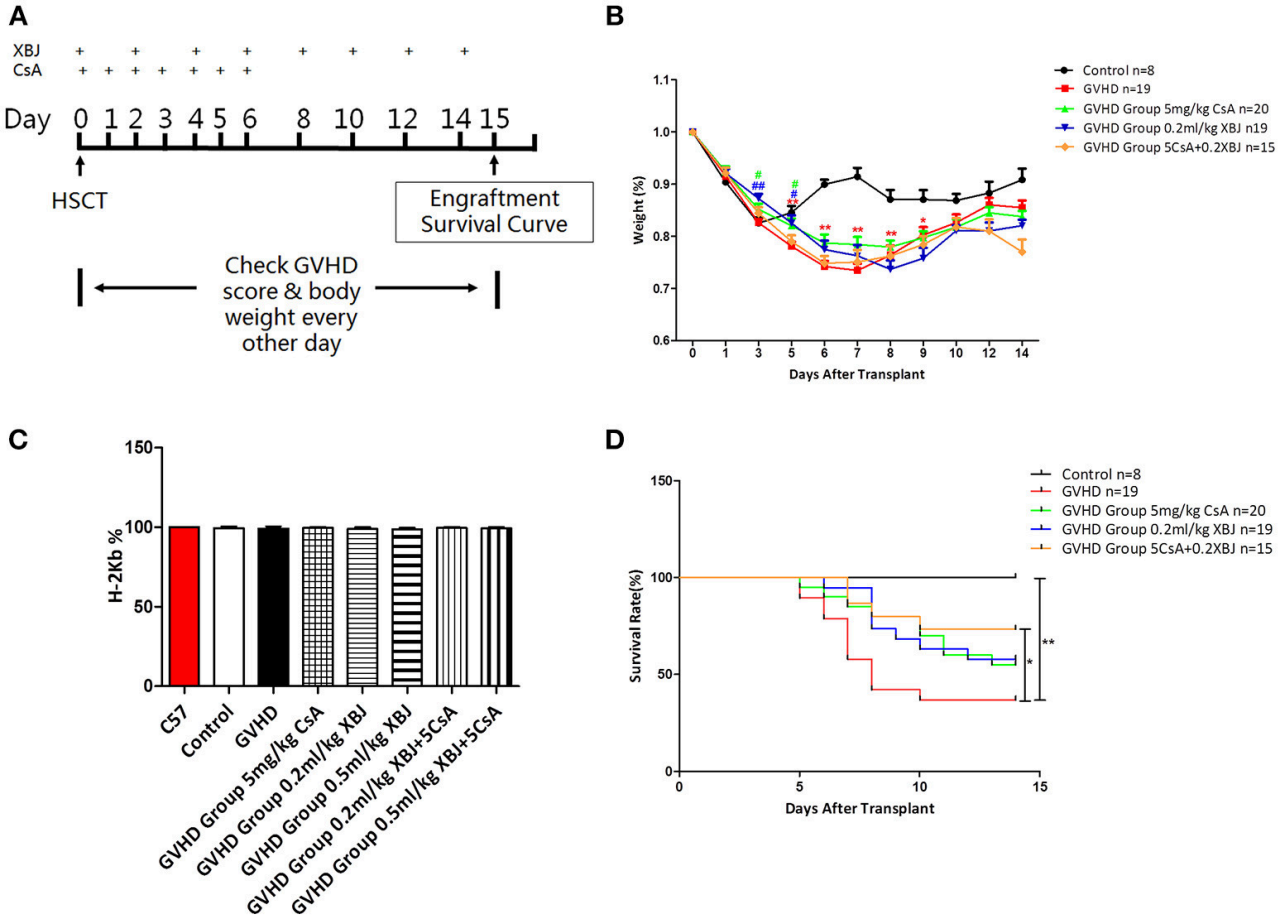
Combination of XBJ and CsA prevented acute GVHD and improved survival in a murine aGVHD model. **(A)** Experimental scheme. **(B)** Body weight change after the transplant in different groups of mice. **(C)** FACS analysis revealed the percentage of MNCs derived from donors (H-2 Kb positive) 14 days after HSCT. **(D)** Survival curve of different groups of mice.

We further tested whether the combination of XBJ and CsA can prevent acute GVHD *in vivo*. The scheme of our in vivo experiments was presented in Figure 9A. Combination of XBJ (0.2 mL/kg) with CsA significantly improved the survival of aGVHD mice (Figure 9D) without compromising the engraftment of hematopoietic stem/progenitor cells from donors (Figure 9C). The combination of XBJ and CsA prevented weight loss at the early stage of aGVHD and showed the trend of improving survival (Figures 9B,D). XBJ treatment and the combination of XBJ and CsA also improved GVHD score in the treatment groups (Supplementary Figure 2). Overall, network pharmacology can guide the development of novel regimens to prevent GVHD. The new integrative medicine presented here sheds light on preventing aGVHD.

## Discussion

In this study, we used network pharmacology to guide the design of a novel integrative regimen in GVHD prophylaxis. Network pharmacology analysis anticipated that XBJ intervenes aGVHD systematically rather than targeting one pathway. The combination of CsA and XBJ was effective in rescuing mice subjected to lethal GVHD and is potentially superior to either drug alone. This was partially achieved by preventing NF-κB nuclear translocation, attenuating inflammation, and maintaining the viability of cells.

### Network Pharmacology Analysis Predicted That Key Regulators in Acute GVHD Are XBJ Targets

Empowered with network pharmacology analysis, we aimed to identify a Chinese medicine to target aGVHD systematically. Four hundred key regulators in acute GVHD were identified, using literature mining, online resources (genecards.com) and IPA. Our analysis predicted TNF, IL-6, IFNγ, and IL-1β are among the top 10 important nodes in the aGVHD molecular network (genecards.com). Network pharmacology analysis and literature search revealed that 38 compounds detected in XBJ ingredients may benefit patients receiving allo-HSCT therapy and those suffering from aGVHD, including active compounds in XBJ that were detected in serum such as paeoniflorin and carthamin yellow (Huang et al., 2011; Li et al., 2016a; Zuo et al., 2017a,b). Our analysis suggested XBJ may prevent aGVHD at different phases. It may ease the tissue injuries caused by the conditioning chemotherapy and radiation-therapy in the conditioning stage. XBJ also prevents effector T cells activation and cell apoptosis in target tissues in the later stage of aGVHD. In addition, XBJ may prevent cytokine storm and inflammation in all stages of aGVHD. Thus, XBJ may prevent aGVHD comprehensively.

### Network Pharmacology Analysis to Compare Danshen and XBJ Targets in aGVHD

Danshen injection has been shown to prevent aGVHD in rats (Yan et al., 2009). It is believed that its major active ingredient, Danshensu, contributed to this phenomenon. Network pharmacology analysis revealed 49 aGVHD related targets can be regulated by 12 compounds uniquely derived from Danshen. In contrast, IPA analysis foretold that 38 compounds in XBJ regulate 110 aGVHD genes. In addition, IPA analysis also revealed that CsA, a commonly used immunosuppressor to prevent aGVHD, shared many aGVHD targets with XBJ (Supplementary Figure 1). Thus, we hypothesize XBJ may enhance aGVHD prevention when combined with CsA.

### XBJ Dose-Dependently Inhibits Platelet Aggregation

In two different experiments, XBJ dose-dependently inhibited platelet aggregation, confirming its anti-clotting effect *in vivo* (Figure 3). Blood clotting directly threatens the survival of allo-HSCT patients and induces inflammation that worsens aGVHD (Ge et al., 2018). XBJ may prevent aGVHD partially through inhibiting clotting and clotting induced inflammation.

### XBJ Promotes Angiogenesis and May Attenuate Organ Damages After Allo-HSCT

XBJ can improve organ injury and tissue damage in patients and animal models (Gao et al., 2015; Yuxi et al., 2017). This might partially due to its effect on angiogenesis. We demonstrated that XBJ promoted tube-formation in EA.hy926 human endothelial cells which could be important in attenuating aGVHD in patients after allo-HSCT. Chemotherapy and radiation conditioning cause damages in different organs and tissues, including blood vessels in the bone marrow and other organs (Hingorani et al., 2015; Perkey and Maillard, 2018). Thus, XBJ may prevent aGVHD through preventing vessel damage and enhancing vessel repair after allo-HSCT.

### XBJ Inhibits NF-κB Signaling in Mouse Macrophages Without Compromising the Viability

Macrophage activation is a key step for aGVHD initiation (Choi et al., 2010b). Upon LPS stimulation, NF-κB translocates into the nucleus to activate inflammation signaling in macrophages. Later, activated macrophages and neutrophils secrete pro-inflammatory cytokines such as IL-6 and TNF-α which may result in cytokine storm that promotes aGVHD (Zeiser et al., 2016; Perkey and Maillard, 2017). We found that XBJ inhibited NF-κB nuclear translocation, indicating XBJ may prevent macrophage activation and normalize cytokine production to prevent aGVHD. On the other hand, it protects the innate immune system through enhancing the viability of macrophages (Figure 7A), thereby preventing fatal infections in allo-HSCT patients. Our result is consistent with the report that low-dose XBJ rescued mice from lethal irradiation while protected HSPCs and MNCs from irradiation-induced apoptosis (Li et al., 2014). Overall, XBJ may prevent GVHD by reducing tissue injuries and preventing overactivation of the immune system.

### Combination of XBJ and CsA Prevented Acute GVHD and Improved Survival in a Murine aGVHD Model

Our *in vitro* experiments and network pharmacology analysis suggest that XBJ may prevent aGVHD systematically rather than affecting a single pathway. We hypothesized that combination of XBJ and CsA is superior to either agent alone in preventing aGVHD. Our hypothesis was supported by results from a lethal aGVHD model (Figure 9), suggesting systematic intervention by combining Chinese medicine with standard management may yield better results in the clinic. Westerhoff et al. (2015) pointed out that systematic interventions are preferred to treat system biology diseases. Reflecting the perspective of system biology, Chinese medicine has great potential for treating complex diseases such as sepsis and GVHD. Combination of a systematic approach with targeted therapies may improve clinical outcomes in GVHD prophylaxis.

### Potential Mechanism of XBJ in Preventing GVHD

We believe that XBJ prevents GVHD by protecting organs, controlling inflammation, and preventing over-activation of effector T cells. XBJ was well known for its effect on preventing organ failure in sepsis and septic shock patients (Gao et al., 2015; Yuxi et al., 2017). A clinical study showed XBJ significantly improved the lung function and the survival of acute respiratory distress syndrome patients (Yue et al., 2015). Preclinical research also confirmed its important role in preventing/healing organ injuries (Xu et al., 2017; Tian et al., 2018). Caffeic acid, a compound detected in XBJ injection (Supplementary Table 2), improved liver injury after liver transplantation (Mu et al., 2018). It attenuated ROS production, reduced productions of pro-inflammation cytokines (TNFα and IL-1β etc.), and improved microcirculation in a rat liver transplant model, indicating caffeic acid in XBJ may contributed to tissue protection. In addition, salvianolic acids in XBJ, which are derived from Danshen may also contributed to tissue protection (Supplementary Table 2). Chen et al. reported that T541, a compound Chinese medicine, containing 40% of total salvianolic acids, ameliorated brain injury caused by delayed tissue-type plasminogen activator (tPA) in a rat thrombosis model (Chen et al., 2018a). Whether salvianolic acids, caffeic acid, and other major compounds in XBJ can prevent aGVHD synergistically is still an open question.

We found that XBJ promoted regulatory T cell differentiation and inhibited T helper cell differentiation in our previous study (Chen et al., 2018b), indicating XBJ may attenuate effector T cell activation to prevent tissue injuries. Our and other studies showed XBJ normalizes the expression of pro-inflammatory cytokine expression in animal models of sepsis and sepsis patients (Jiang et al., 2013; Chen et al., 2018b). These evidence suggested that XBJ may prevent GVHD systemically by preventing cytokine storm, halting effector T cell activation and promoting wound healing.

### Integrative Medicine and GVHD Prophylaxis

More evidence have shown that multi-target approaches is more effective in treating complex diseases such as sepsis, cancer and GVHD than the single target approach (Csermely et al., 2005; Yang et al., 2008; Vazquez, 2009; Motter, 2010). Multiple targets oriented combination therapy is also becoming a trend for GVHD prevention and treatment (Zeiser et al., 2016). It is believed that relieving tissue injuries contribute to preventing GVHD (Zeiser et al., 2016; Perkey and Maillard, 2018). However, current regimens of GVHD prophylaxis did not offer solutions for tissue injuries caused by conditioning regimens, cancer therapies and aGVHD. Side-effects of CsA and other chemotherapy drugs also compromise the health of allo-HSCT recipients, increasing the risk of organ failure and infection. Compound Chinese medicine provide a potential solution for these complications. XBJ, a Chinese medicine injection that activates circulation and removing stasis, attenuated aGVHD and improved survival in a lethal aGVHD model, indicating Chinese medicine may benefit allo-HSCT recipients in the clinic. The mechanism of XBJ in alleviating tissue injuries is partially through anti-clotting, promoting angiogenesis, improving microcirculation and anti-inflammation (Figures 6–8; Jiang et al., 2013; Chen et al., 2018a; Mu et al., 2018). HMGB1 might be a XBJ target in preventing tissue injury and aGVHD (Wang et al., 2015; Chen et al., 2016). These results indicated the integrative medicine may shed light on preventing GVHD.

### The Novelty of Integrating Network Pharmacology Into the Development of Novel Regimens to Prevent GVHD

Network Pharmacology revolutionized the development of novel therapies for human diseases (Barabasi et al., 2011; Leung et al., 2013; Li and Zhang, 2013). Its application in preventing GVHD is limited. This work explored the potential of utilizing network pharmacology to guide the development of a novel integrative regimen to prevent GVHD and provided supporting evidence for the future application of network pharmacology in GVHD prophylaxis.

### Future Directions

Preventing cytokine storm, organ injuries, and inflammation contribute to aGVHD prophylaxis. How does the combination of XBJ and CsA affect cytokine production in macrophages and in the animal model remains to be investigated. Given the critical role of gut microbiota in aGVHD development (Jenq et al., 2012; Perkey and Maillard, 2017), the influence of XBJ on gut microbiota and intestine tissue after allo-HSCT should be explored in animal models and patients.

## Conclusion

In summary, combining network pharmacology with experimental pharmacology approaches may effectively promote preclinical and clinical research on complex diseases such as aGVHD prophylaxis. Integrative medicine, which adapts a holistic approach, may provide novel solutions for GVHD prevention and treatment.

## Author Contributions

ML, YF, GP, and YZ designed the study and developed the methodologies. ML, ZZ, XW, YF, and GP conducted research. ZZ, HL, MW, YW, GF, XG, and YZ analyzed the data, and contributed critical reagents. ML, ZZ, YF, HL, and YZ wrote the manuscript.

### Conflict of Interest Statement

The authors declare that the research was conducted in the absence of any commercial or financial relationships that could be construed as a potential conflict of interest.

## Supplementary Material

Supplementary Figure 1 Comparison of top six IPA-predicted chemical drugs that regulate predicted XBJ and Danshen targets. A. Relationships of XBJ regulated proteins and top six drugs that can interfere with their activities. The top six predicted drugs of XBJ targets are: lipopolysaccharide, cyclosporin A, curcumin, SB203580, phorbol myristate acetate and N-acetyl-L-cysteine. B. Relationships of Danshen regulated proteins and top six drugs predicted by IPA that can interfere with their activities. The top 6 drugs of Danshen targets are: simvastatin, epigallocatechin-gallate, LY294002, ethanol, PD98059 and curcumin.

Supplementary Figure 2 GVHD scores in different groups of mice after the transplantation. ^*^: The significant difference between Control and GVHD group; #: the significant difference between each treatment group and GVHD group; ▴: the significant difference between the combination of XBJ and CsA, and XBJ or CsA alone. ^*^: *P* < 0.05; ^**^: *P* < 0.01; #: *P* < 0.05; ##: *P* < 0.01.

Supplementary Figure 3 NF-κB nuclear translocation was detected by an Operetta high-content florescent imaging system (Perkin Elmer) in RAW264.7 cells. After LPS stimulation for 30 min, cells were treated with indicated doses of XBJ and DEX for 12 h before the fixation, antibody staining and imaging. XBJ was diluted in 1/20 and 1/200. Dexamethasone (DEX) was used as a positive control in the experiment and 10^−3^, 10^−4^, and 10^−5^ mM of DEX were used to treat 264.7 cells.
