# Supplementary figures and images for: Network Pharmacology-Guided Development of a Novel Integrative Regimen to Prevent Acute Graft-vs.-Host Disease
Source: Front Pharmacol. 2018 Dec 13;9:1440. doi: 10.3389/fphar.2018.01440 (PMC6300759; doi:10.3389/fphar.2018.01440)

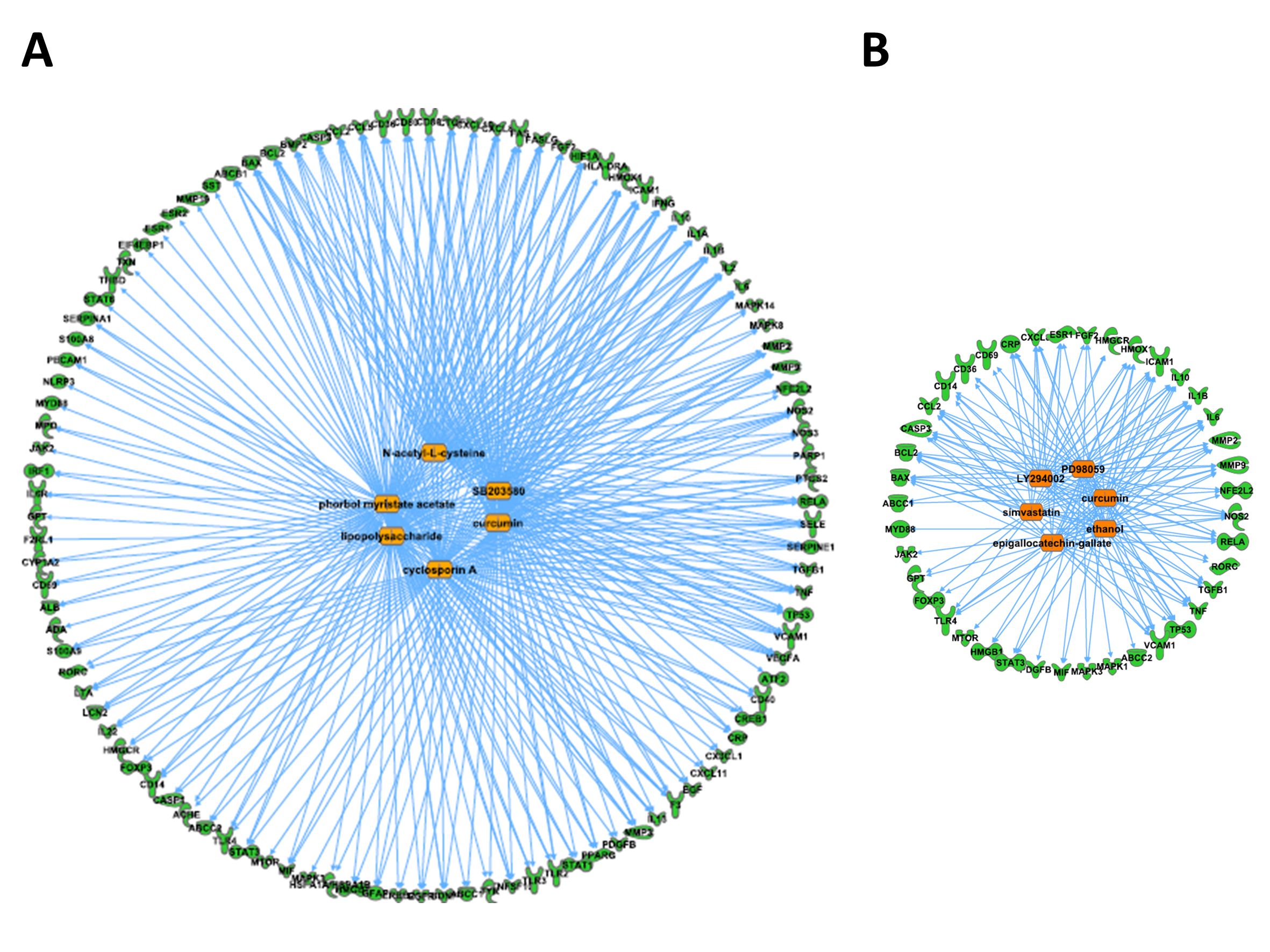

Supplement: Supplementary Figure 1 — Comparison of top six IPA-predicted chemical drugs that regulate predicted XBJ and Danshen targets. A. Relationships of XBJ regulated proteins and top six drugs that can interfere with their activities. The top six predicted drugs of XBJ targets are: lipopolysaccharide, cyclosporin A, curcumin, SB203580, phorbol myristate acetate and N-acetyl-L-cysteine. B. Relationships of Danshen regulated proteins and top six drugs predicted by IPA that can interfere with their activities. The top 6 drugs of Danshen targets are: simvastatin, epigallocatechin-gallate, LY294002, ethanol, PD98059 and curcumin. [file Image_1.tif]

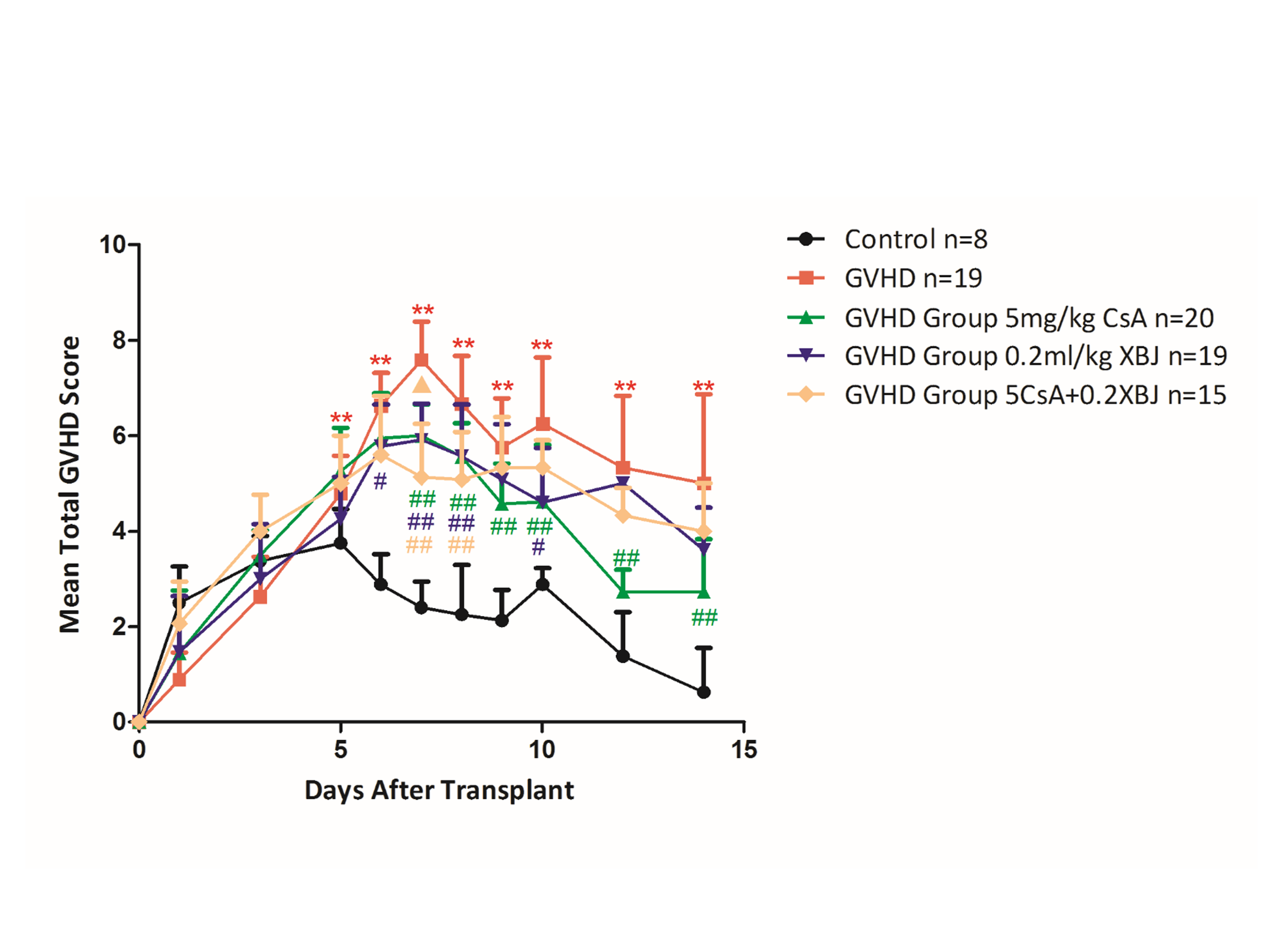

Supplement: Supplementary Figure 2 — GVHD scores in different groups of mice after the transplantation. *: The significant difference between Control and GVHD group; #: the significant difference between each treatment group and GVHD group; ▴: the significant difference between the combination of XBJ and CsA, and XBJ or CsA alone. *: P < 0.05; **: P < 0.01; #: P < 0.05; ##: P < 0.01. [file Image_2.tif]

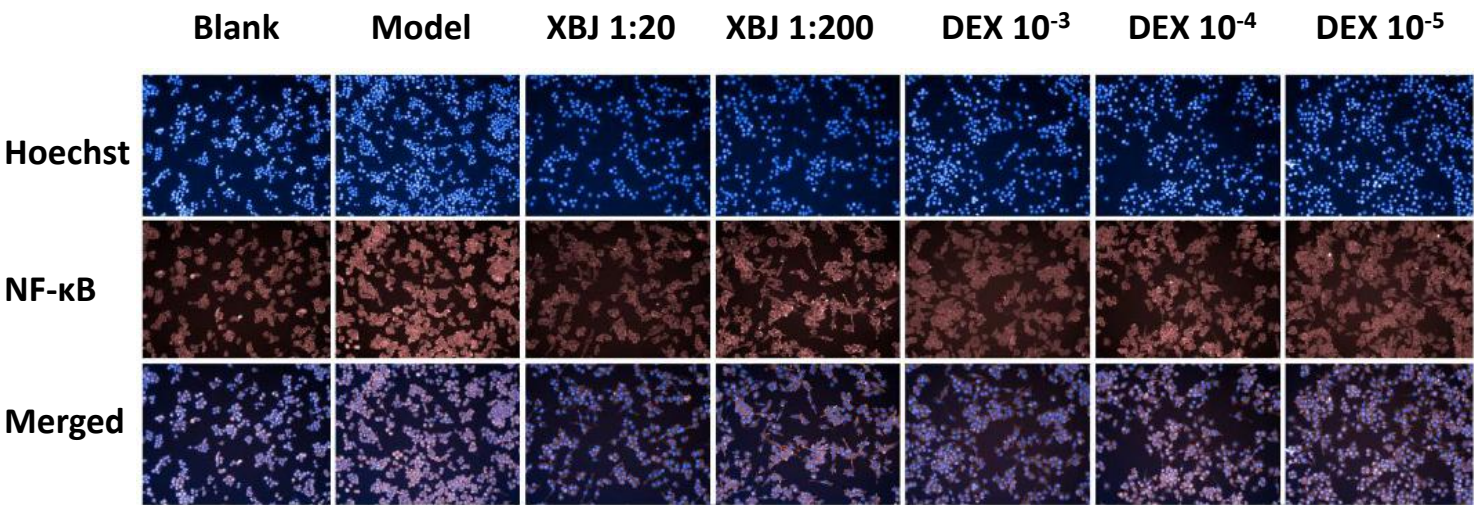

Supplement: Supplementary Figure 3 — NF-κB nuclear translocation was detected by an Operetta high-content florescent imaging system (Perkin Elmer) in RAW264.7 cells. After LPS stimulation for 30 min, cells were treated with indicated doses of XBJ and DEX for 12 h before the fixation, antibody staining and imaging. XBJ was diluted in 1/20 and 1/200. Dexamethasone (DEX) was used as a positive control in the experiment and 10−3, 10−4, and 10−5 mM of DEX were used to treat 264.7 cells. [file Image_3.pdf]
