# Supplementary material for: Network Pharmacology-Guided Development of a Novel Integrative Regimen to Prevent Acute Graft-vs.-Host Disease
Source: Front Pharmacol. 2018 Dec 13;9:1440. doi: 10.3389/fphar.2018.01440 (PMC6300759; doi:10.3389/fphar.2018.01440)
Supplement: Supplementary file 4 [file Table_1.PDF]

Supplemental Table 1. Acute GVHD target list					
ID	Symbol	Entrez Gene Name	Location	Type(s)	Drug(s)
ABCB1	ABCB1	ATP binding cassette subfamily B member 1	Plasma Membrane	transporter	dofequidar, tariquidar, OC 144-093, valspodar
ABCC1	ABCC1	ATP binding cassette subfamily C member 1	Plasma Membrane	transporter	sulfinpyrazone
ABCC2	ABCC2	ATP binding cassette subfamily C member 2	Plasma Membrane	transporter	sulfinpyrazone
ACE	ACE	angiotensin I converting enzyme	Plasma Membrane	peptidase	pentopril, quinaprilat, perindoprilat, amlodipine/perindopril, benazeprilat, trandolaprilat, angiotensin-converting enzyme inhibitor, aspirin/lisinopril, amlodipine/benazepril, hydrochlorothiazide/lisinopril, benazepril, enalapril, perindopril, captopril, cilazapril, enalapril/felodipine, hydrochlorothiazide/moexipril, benazepril/hydrochlorothiazide, hydrochlorothiazide/quinapril, fosinopril/hydrochlorothiazide, captopril/hydrochlorothiazide, enalapril/hydrochlorothiazide, ramipril, ramiprilat, moexipril, quinapril, lisinopril, enalaprilat, trandolapril,

					trandolapril/verapamil, diltiazem/enalapril, fosinopril
ACHE	ACHE	acetylcholinesterase (Cartwright blood group)	Plasma Membrane	enzyme	ZT-1, ladostigil, terestigmine, acetylcholinesterase inhibitor, donepezil/memantine, caracemide, donepezil/solifenacin, atropine/edrophonium, BW 284 C 51, neostigmine, 2,3 butanedione monoxime, echothiophate, tacrine, edrophonium, isoflurophate, pyridostigmine, demecarium, ambenonium, donepezil, pralidoxime, rivastigmine, gallamine triethiodide, thiamine, huperzine A, physostigmine, galanthamine
ACTC1	ACTC1	actin, alpha, cardiac muscle 1	Cytoplasm	enzyme	
ADA	ADA	adenosine deaminase	Cytoplasm	enzyme	pentostatin, aspirin/dipyridamole/telmisartan, cyclophosphamide/pentostatin/rituximab, pentostatin/rituximab, alemtuzumab/pentostatin, adenosine deaminase inhibitor, dipyridamole, aspirin/dipyridamole, vidarabine
ADCY10	ADCY10	adenylate cyclase 10	Cytoplasm	enzyme	
AGTR2	AGTR2	angiotensin II receptor type 2	Plasma Membrane	G-protein coupled receptor	

ALB	ALB	albumin	Extracellular Space	transporter	gadofosveset trisodium, aldoxorubicin, GSK3128349
ALPP	ALPP	alkaline phosphatase, placental	Plasma Membrane	phosphatase	
AMBP	AMBP	alpha-1-microglobulin/bikunin precursor	Extracellular Space	transporter	
ANGPT1	ANGPT1	angiopoietin 1	Extracellular Space	growth factor	trebananib
ANGPT2	ANGPT2	angiopoietin 2	Extracellular Space	growth factor	trebananib, CVX-060, vanucizumab
ANPEP	ANPEP	alanyl aminopeptidase, membrane	Plasma Membrane	peptidase	CNGRC peptide-TNF alpha conjugate
ANXA2	ANXA2	annexin A2	Plasma Membrane	other	
ANXA5	ANXA5	annexin A5	Plasma Membrane	transporter	
APCS	APCS	amyloid P component, serum	Extracellular Space	other	
APEX1	APEX1	apurinic/apyrimidinic endodeoxyribonuclease 1	Nucleus	enzyme	
APOB	APOB	apolipoprotein B	Extracellular Space	transporter	mipomersen
APOE	APOE	apolipoprotein E	Extracellular Space	transporter	
APOH	APOH	apolipoprotein H	Extracellular Space	transporter	
AREG	AREG	amphiregulin	Extracellular Space	growth factor	
B2M	B2M	beta-2-microglobulin	Plasma Membrane	transmembrane receptor	4'-iodo-4'-deoxydoxorubicin
BAX	BAX	BCL2 associated X, apoptosis regulator	Cytoplasm	transporter	

BCL2	BCL2	BCL2, apoptosis regulator	Cytoplasm	transporter	oblimersen, rasagiline, (-)-gossypol, BCL-2 blocker, navitoclax, gemcitabine/paclitaxel, bortezomib/paclitaxel, venetoclax, paclitaxel/trastuzumab, paclitaxel/pertuzumab/trastuzumab, lapatinib/paclitaxel, doxorubicin/paclitaxel, epirubicin/paclitaxel, paclitaxel/ramucirumab, paclitaxel/topotecan, BCL201, S 055746, APG-1252, rituximab/venetoclax, paclitaxel/rituximab, paclitaxel
BCL2A1	BCL2A1	BCL2 related protein A1	Cytoplasm	other	
BCL2L1	BCL2L1	BCL2 like 1	Cytoplasm	other	
BDNF	BDNF	brain derived neurotrophic factor	Extracellular Space	growth factor	
BIRC5	BIRC5	baculoviral IAP repeat containing 5	Cytoplasm	other	gataparsen, EZN 3042
BMP2	BMP2	bone morphogenetic protein 2	Extracellular Space	growth factor	
BMP6	BMP6	bone morphogenetic protein 6	Extracellular Space	growth factor	
BMP7	BMP7	bone morphogenetic protein 7	Extracellular Space	growth factor	
BPI	BPI	bactericidal/permeability-increasing protein	Plasma Membrane	transporter	
BSG	BSG	basigin (Ok	Plasma	transporter	

		blood group)	Membrane		
CABIN1	CABIN1	calcineurin binding protein 1	Nucleus	other	
CALCA	CALCA	calcitonin related polypeptide alpha	Plasma Membrane	other	
CAMP	CAMP	cathelicidin antimicrobial peptide	Cytoplasm	other	
CASP1	CASP1	caspase 1	Cytoplasm	peptidase	caspase 1 inhibitor
CASP3	CASP3	caspase 3	Cytoplasm	peptidase	caspase 3 inhibitor, emricasan
CASP8	CASP8	caspase 8	Nucleus	peptidase	
CAT	CAT	catalase	Cytoplasm	enzyme	fomepizole
CCL11	CCL11	C-C motif chemokine ligand 11	Extracellular Space	cytokine	bertilimumab
CCL2	CCL2	C-C motif chemokine ligand 2	Extracellular Space	cytokine	mimosine
CCL5	CCL5	C-C motif chemokine ligand 5	Extracellular Space	cytokine	
CCR1	CCR1	C-C motif chemokine receptor 1	Plasma Membrane	G-protein coupled receptor	
CCR2	CCR2	C-C motif chemokine receptor 2	Plasma Membrane	G-protein coupled receptor	PF-4136309
CCR3	CCR3	C-C motif chemokine receptor 3	Plasma Membrane	G-protein coupled receptor	
CCR5	CCR5	C-C motif chemokine receptor 5 (gene/pseudogene)	Plasma Membrane	G-protein coupled receptor	maraviroc, vicriviroc, ancriviroc
CCR6	CCR6	C-C motif chemokine receptor 6	Plasma Membrane	G-protein coupled receptor	
CCR7	CCR7	C-C motif chemokine	Plasma Membrane	G-protein coupled	

		receptor 7		receptor	
CCR9	CCR9	C-C motif chemokine receptor 9	Plasma Membrane	G-protein coupled receptor	
CD14	CD14	CD14 molecule	Plasma Membrane	transmembrane receptor	
CD163	CD163	CD163 molecule	Plasma Membrane	transmembrane receptor	
CD2	CD2	CD2 molecule	Plasma Membrane	transmembrane receptor	alefacept, siplizumab
CD28	CD28	CD28 molecule	Plasma Membrane	transmembrane receptor	
CD36	CD36	CD36 molecule	Plasma Membrane	transmembrane receptor	
CD38	CD38	CD38 molecule	Plasma Membrane	enzyme	daratumumab, isatuximab
CD4	CD4	CD4 molecule	Plasma Membrane	transmembrane receptor	
CD40	CD40	CD40 molecule	Plasma Membrane	transmembrane receptor	dacetuzumab, CFZ533, FFP104, SEA-CD40, APX005M
CD40LG	CD40LG	CD40 ligand	Extracellular Space	cytokine	
CD44	CD44	CD44 molecule (Indian blood group)	Plasma Membrane	other	anti-CD44v7 antibody
CD55	CD55	CD55 molecule (Cromer blood group)	Plasma Membrane	other	
CD79A	CD79A	CD79a molecule	Plasma Membrane	transmembrane receptor	
CD80	CD80	CD80 molecule	Plasma Membrane	transmembrane receptor	abatacept, belatacept, abatacept/methotrexate
CD86	CD86	CD86	Plasma	transmemb	abatacept, belatacept,

		molecule	Membrane	rane receptor	abatacept/methotrexate
CD8A	CD8A	CD8a molecule	Plasma Membrane	other	
CDH5	CDH5	cadherin 5	Plasma Membrane	other	
CDKN1A	CDKN1A	cyclin dependent kinase inhibitor 1A	Nucleus	kinase	
CIITA	CIITA	class II major histocompatibility complex transactivator	Nucleus	transcription regulator	
CLEC7A	CLEC7A	C-type lectin domain containing 7A	Plasma Membrane	transmembrane receptor	
CLU	CLU	clusterin	Cytoplasm	other	
CR1	CR1	complement C3b/C4b receptor 1 (Knops blood group)	Plasma Membrane	transmembrane receptor	
CREB1	CREB1	cAMP responsive element binding protein 1	Nucleus	transcription regulator	
CRP	CRP	C-reactive protein	Extracellular Space	other	
CSF1	CSF1	colony stimulating factor 1	Extracellular Space	cytokine	
CSF2	CSF2	colony stimulating factor 2	Extracellular Space	cytokine	
CSF3	CSF3	colony stimulating factor 3	Extracellular Space	cytokine	
CST3	CST3	cystatin C	Extracellular	other	

			Space		
CTGF	CTGF	connective tissue growth factor	Extracellular Space	growth factor	FG-3019
CTLA4	CTLA4	cytotoxic T-lymphocyte associated protein 4	Plasma Membrane	transmembrane receptor	ipilimumab, ticilimumab, ipilimumab/nivolumab, anti CTLA-4 antibody, BMS-986218
CTSD	CTSD	cathepsin D	Cytoplasm	peptidase	
CTSK	CTSK	cathepsin K	Cytoplasm	peptidase	cathepsin K inhibitor, odanacatib
CX3CL1	CX3CL1	C-X3-C motif chemokine ligand 1	Extracellular Space	cytokine	
CX3CR1	CX3CR1	C-X3-C motif chemokine receptor 1	Plasma Membrane	G-protein coupled receptor	
CXCL10	CXCL10	C-X-C motif chemokine ligand 10	Extracellular Space	cytokine	MDX-1100
CXCL13	CXCL13	C-X-C motif chemokine ligand 13	Extracellular Space	cytokine	
CXCR3	CXCR3	C-X-C motif chemokine receptor 3	Plasma Membrane	G-protein coupled receptor	
CXCR4	CXCR4	C-X-C motif chemokine receptor 4	Plasma Membrane	G-protein coupled receptor	cladribine/cytarabine/filgrastim/idarubicin/plerixafor, POL6326, BL-8040, LY-2510924, burixafor, USL311, PF-06747143, plerixafor
CYBA	CYBA	cytochrome b-245 alpha chain	Cytoplasm	enzyme	
CYP1A1	CYP1A1	cytochrome P450 family 1 subfamily A member 1	Cytoplasm	enzyme	
CYP1A2	CYP1A2	cytochrome P450 family	Cytoplasm	enzyme	

		1 subfamily A member 2			
CYP2B6	CYP2B6	cytochrome P450 family 2 subfamily B member 6	Cytoplasm	enzyme	
CYP3A4	CYP3A4	cytochrome P450 family 3 subfamily A member 4	Cytoplasm	enzyme	cobicistat, cobicistat/elvitegravir/emtricitabine/tenofovir disoproxil, atazanavir/cobicistat, cobicistat/darunavir, ketoconazole
CYP3A5	CYP3A5	cytochrome P450 family 3 subfamily A member 5	Cytoplasm	enzyme	cobicistat, cobicistat/elvitegravir/emtricitabine/tenofovir disoproxil, atazanavir/cobicistat, cobicistat/darunavir
DES	DES	desmin	Cytoplasm	other	
DHFR	DHFR	dihydrofolate reductase	Nucleus	enzyme	pyrimethamine, trimethoprim, iclaprim, proguanil, methotrexate/ofatumumab, methotrexate/sirolimus/tacrolimus, pralatrexate, abatacept/methotrexate, infliximab/methotrexate, methotrexate/rituximab, golimumab/methotrexate, cisplatin/doxorubicin/methotrexate, cytarabine/methotrexate, cisplatin/doxorubicin/ifosfamide/methotrexate, methotrexate/rituximab/temozolomide, ifosfamide/methotrexate, bevacizumab/pemetrexed, cytarabine/dexamethasone/methotrexate, leucovorin/methotrexate, cyclophosphamide/methot

					rexate, adalimumab/methotrexate, gemcitabine/pemetrexed, epinephrine/methotrexate, dihydrofolate reductase inhibitor, ABTL0812, methotrexate/tofacitinib, etanercept/methotrexate, methotrexate/ustekinumab, baricitinib/methotrexate, methotrexate/tocilizumab, methotrexate/sarilumab, cyclophosphamide/methotrexate/trastuzumab, methotrexate, atovaquone/proguanil, sulfisoxazole, triamterene, folic acid, trimetrexate, pemetrexed, talotrexin
DRD2	DRD2	dopamine receptor D2	Plasma Membrane	G-protein coupled receptor	paliperidone, risperidone, buspirone, carbidopa/entacapone/levodopa, bifeprunox, iloperidone, blonanserin, asenapine, pardoprunox, ocaperidone, abaperidone, methotrimeprazine, fluspirilene, SLV-314, cariprazine, rotigotine, acetophenazine, BIM 23A760, sultopride, zuclopenthixol, thioproperazine, lurasidone, opipramol, paliperidone palmitate, brexpiprazole, pipothiazine, benperidol, carbidopa/levodopa, chloropromazine, domperidone, metoclopramide, sulpiride, meloxicam, amantadine, flupenthixol, chlorprothixene,

					trifluoperazine, dexamethasone/olanzapine, haloperidol/octreotide, fluphenazine, pimozide, clozapine, haloperidol, fluoxetine/olanzapine, fluphenazine decanoate, thiothixene, amitriptyline/perphenazine, haloperidol decanoate, molindone, trimethobenzamide, fluphenazine enanthate, loxapine, perphenazine, promazine, prochlorperazine, triflupromazine, quetiapine, pramipexole, olanzapine, remoxipride, lisuride, sertindole, cabergoline, ziprasidone, mesoridazine, thioridazine, aripiprazole, ropinirole, dihydroergocryptine, dihydroergotamine, bromocriptine, apomorphine, pergolide, dopamine, droperidol, thiethylperazine, droperidol/fentanyl, L-dopa
EDN1	EDN1	endothelin 1	Extracellular Space	cytokine	
EDN3	EDN3	endothelin 3	Extracellular Space	other	
EDNRA	EDNRA	endothelin receptor type A	Plasma Membrane	transmembrane receptor	bosentan, avosentan, clazosentan, ambrisentan, sitaxsentan, zibotentan, SB 234551, TBC 3214, BSF 302146, macitentan, ambrisentan/tadalafil, fandosentan, atrasentan
EGF	EGF	epidermal	Extracellular	growth	

		growth factor	Space	factor	
EGFR	EGFR	epidermal growth factor receptor	Plasma Membrane	kinase	cetuximab, AEE 788, panitumumab, BMS-599626, varlitinib, tesevatinib, BMS-690514, bevacizumab/erlotinib, afatinib, nimotuzumab, necitumumab, BIBX 1382BS, icotinib, MP 412, sapitinib, cetuximab/irinotecan, lapatinib/pazopanib, irinotecan/panitumumab, erlotinib/vismodegib, erlotinib/gemcitabine, JNJ-26483327, lapatinib/letrozole, capecitabine/lapatinib, bevacizumab/panitumumab, bevacizumab/cetuximab, EGFR tyrosine kinase inhibitor, capecitabine/erlotinib, lapatinib/paclitaxel, rociletinib, brigatinib, MEHD7945A, cabozantinib/erlotinib, osimertinib, poziotinib, olmutinib, sym004, IMGN289, EGFR antisense DNA, pyrotinib, EGF816, naquotinib, selatinib, 18F-PEG6-IPQA, lapatinib/trastuzumab, ABT-414, docetaxel/lapatinib, anti-EGFR monoclonal antibody GT-MAB 5.2-GEX, anti-EGFR antibody, AZD3759, avitinib, PF-06747775,

					LY3164530, RG 14620, TAK-285, SAR103168, PF-06459988, bis-tyrphostin, tyrphostin A48, tyrphostin A30, RG 13022, RM-1929, hemay022, hemay020, pirotinib, ABBV-221, AC0010MA, afatinib/cetuximab, dabrafenib/panitumumab, BGB-283, cetuximab/panitumumab, erlotinib/gefitinib, dabrafenib/panitumumab/trametinib, AP32788, everolimus/gefitinib, depatuxizumab, EGFR monoclonal antibody, sym013, theliatinib, doxorubicin-loaded EGFR-targeting nanocells, HS-10296, canertinib, gefitinib, YH25448, BPI-15086, neratinib, PD 153035, pelitinib, SKLB1028, lapatinib, vandetanib, erlotinib
EGR1	EGR1	early growth response 1	Nucleus	transcription regulator	
ELANE	ELANE	elastase, neutrophil expressed	Extracellular Space	peptidase	2-O,3-O-desulfated heparin, SERPINA1
ELN	ELN	elastin	Extracellular Space	other	
ENO2	ENO2	enolase 2	Cytoplasm	enzyme	
EPO	EPO	erythropoietin	Extracellular Space	cytokine	
ESR1	ESR1	estrogen receptor 1	Nucleus	ligand-dependent nuclear receptor	17-alpha-ethinylestradiol, fulvestrant, beta-estradiol, estradiol 17beta-cypionate, estriol, estrone, estradiol valerate,

					estrone sulfate, mestranol, CHF-4227, bazedoxifene, estradiol valerate/testosterone enanthate, TAS-108, ethynodiol diacetate, ethinyl estradiol/ethynodiol diacetate, estradiol acetate, esterified estrogens, estradiol cypionate/medroxyprogesterone acetate, estradiol/norethindrone acetate, estradiol cypionate/testosterone cypionate, synthetic conjugated estrogens, B, etonogestrel, CC8490, MITO-4509, cyproterone acetate/ethinyl estradiol, ethinyl estradiol/etonogestrel, pipendoxifene, chlorotrianisene, megestrol acetate/tamoxifen, sulindac/tamoxifen, sulindac/toremifene, raloxifene/sulindac, F18 16-alpha-fluoroestradiol, ARN-810, Z-endoxifen, goserelin/tamoxifen, raloxifene/teriparatide, AZD9496, RAD1901, SRN-927, fulvestrant/palbociclib, anastrozole/tamoxifen, fulvestrant/letrozole/tamoxifen, anastrozole/exemestane/fulvestrant, anastrozole/goserelin/tamoxifen,

					anastrozole/fulvestrant/tamoxifen, exemestane/fulvestrant, fulvestrant/letrozole, letrozole/tamoxifen, exemestane/tamoxifen, exemestane/fulvestrant/letrozole/tamoxifen, anastrozole/exemestane/fulvestrant/tamoxifen, anastrozole/fulvestrant/goserelin/tamoxifen, exemestane/fulvestrant/tamoxifen, exemestane/fulvestrant/goserelin/letrozole/tamoxifen, anastrozole/fulvestrant, fulvestrant/pertuzumab/trastuzumab, pertuzumab/tamoxifen/trastuzumab, selective estrogen receptor modulator, desogestrel/ethinyl estradiol, drospirenone/ethinyl estradiol, ethinyl estradiol/norelgestromin, ethinyl estradiol/norethindrone, ethinyl estradiol/levonorgestrel, ethinyl estradiol/norgestrel, ethinyl estradiol/norgestimate, diethylstilbestrol, ospemifene, toremifene, tamoxifen, raloxifene, everolimus/tamoxifen, H3B-6545, arzoxifene, clomiphene, abemaciclib/fulvestrant, estramustine phosphate,

					diethylstilbestrol diphosphate, 4-hydroxytamoxifen, dienestrol, acolbifene, estramustine, medroxyprogesterone acetate, desogestrel, danazol, trilostane, fluoxymesterone, norgestimate, progesterone, S-equol
ESR2	ESR2	estrogen receptor 2	Nucleus	ligand-dependent nuclear receptor	17-alpha-ethinylestradiol, fulvestrant, beta-estradiol, estradiol 17beta-cypionate, estrone, estradiol valerate, CHF-4227, bazedoxifene, estradiol valerate/testosterone enanthate, TAS-108, ethinyl estradiol/ethynodiol diacetate, estradiol acetate, esterified estrogens, estradiol cypionate/medroxyprogesterone acetate, estradiol/norethindrone acetate, estradiol cypionate/testosterone cypionate, synthetic conjugated estrogens, B, CC8490, MITO-4509, megestrol acetate/tamoxifen, sulindac/tamoxifen, sulindac/toremifene, raloxifene/sulindac, goserelin/tamoxifen, raloxifene/teriparatide, anastrozole/tamoxifen, fulvestrant/letrozole/tamoxifen, anastrozole/exemestane/fu

					lvestrant, anastrozole/goserelin/tamoxifen, anastrozole/fulvestrant/tamoxifen, exemestane/fulvestrant, fulvestrant/letrozole, letrozole/tamoxifen, exemestane/tamoxifen, exemestane/fulvestrant/letrozole/tamoxifen, anastrozole/exemestane/fulvestrant/tamoxifen, anastrozole/fulvestrant/goserelin/tamoxifen, exemestane/fulvestrant/tamoxifen, exemestane/fulvestrant/goserelin/letrozole/tamoxifen, anastrozole/fulvestrant, pertuzumab/tamoxifen/trastuzumab, desogestrel/ethinyl estradiol, drospirenone/ethinyl estradiol, ethinyl estradiol/norelgestromin, ethinyl estradiol/norethindrone, ethinyl estradiol/levonorgestrel, ethinyl estradiol/norgestrel, ethinyl estradiol/norgestimate, diethylstilbestrol, ospemifene, toremifene, tamoxifen, prinaberel, raloxifene, everolimus/tamoxifen, arzoxifene, clomiphene, estramustine phosphate, diethylstilbestrol diphosphate, genistein,

					trilostane
F13A1	F13A1	coagulation factor XIII A chain	Extracellular Space	enzyme	
F2	F2	coagulation factor II, thrombin	Extracellular Space	peptidase	enoxaparin, desirudin, dabigatran etexilate, Fibrinogen, ximelagatran, thrombin inhibitor, antithrombin alfa, aspirin/dabigatran etexilate, dabigatran, ulinastatin, aspirin/bivalirudin, argatroban, bivalirudin, lepirudin
F2R	F2R	coagulation factor II thrombin receptor	Plasma Membrane	G-protein coupled receptor	chrysalin, vorapaxar, PAR1 inhibitor, argatroban, bivalirudin
F2RL1	F2RL1	F2R like trypsin receptor 1	Plasma Membrane	G-protein coupled receptor	
F3	F3	coagulation factor III, tissue factor	Plasma Membrane	transmembrane receptor	activated recombinant human factor VII, tisotumab vedotin
F5	F5	coagulation factor V	Extracellular Space	other	drotrecogin alfa, antithrombin alfa
F8	F8	coagulation factor VIII	Extracellular Space	other	drotrecogin alfa, F9
F9	F9	coagulation factor IX	Extracellular Space	peptidase	moroctocog alfa, activated recombinant human factor VII
FABP2	FABP2	fatty acid binding protein 2	Cytoplasm	transporter	
FAS	FAS	Fas cell surface death receptor	Plasma Membrane	transmembrane receptor	
FASLG	FASLG	Fas ligand	Extracellular Space	cytokine	APG101
FCER2	FCER2	Fc fragment of IgE receptor II	Plasma Membrane	transmembrane receptor	

FCGR2A	FCGR2A	Fc fragment of IgG receptor IIa	Plasma Membrane	transmembrane receptor	IgG
FCGR3A	FCGR3A/ FCGR3B	Fc fragment of IgG receptor IIIa	Plasma Membrane	transmembrane receptor	IgG, AFM13
FGA	FGA	fibrinogen alpha chain	Extracellular Space	other	F2
FGF1	FGF1	fibroblast growth factor 1	Extracellular Space	growth factor	pentosan polysulfate
FGF2	FGF2	fibroblast growth factor 2	Extracellular Space	growth factor	pentosan polysulfate, suradista, CP-547632, sucralfate
FGF7	FGF7	fibroblast growth factor 7	Extracellular Space	growth factor	
FKBP1A	FKBP1A	FK506 binding protein 1A	Cytoplasm	enzyme	methotrexate/sirolimus/tacrolimus, everolimus/exemestane, imatinib/sirolimus, cyclophosphamide/sirolimus, cyclosporine A/tacrolimus, cyclosporine A/sirolimus/tacrolimus, everolimus/panobinostat, everolimus/pasireotide, ABI-009, everolimus/sorafenib, everolimus/gefitinib, everolimus/lenvatinib, sirolimus, temsirolimus, tacrolimus, everolimus
FLT3	FLT3	fms related tyrosine kinase 3	Plasma Membrane	kinase	sunitinib, dovitinib, tandutinib, XL999, cabozantinib, amuvatinib, nintedanib, quizartinib, KW 2449, ponatinib, bortezomib/sorafenib, dexamethasone/lenalidomide/sorafenib, bevacizumab/sorafenib, brigatinib, pexidartinib,

					crenolanib, SB-1317, cabozantinib/erlotinib, famitinib, cytarabine/idarubicin/sorafenib, 5-azacytidine/sorafenib, decitabine/sorafenib, FLX925, AKN-028, gilteritinib, 4SC-203, everolimus/sorafenib, FLT3 inhibitor, TTT-3002, docetaxel/nintedanib, 4G8-SDIEM, sorafenib, lestaurtinib, SKLB1028, sorafenib/sulindac/sunitinib, midostaurin
FN1	FN1	fibronectin 1	Extracellular Space	enzyme	ocriplasmin, L19-IL2 monoclonal antibody-cytokine fusion protein
FOS	FOS	Fos proto-oncogene, AP-1 transcription factor subunit	Nucleus	transcription regulator	
FOXP3	FOXP3	forkhead box P3	Nucleus	transcription regulator	
FPR1	FPR1	formyl peptide receptor 1	Plasma Membrane	G-protein coupled receptor	
FTL	FTL	ferritin light chain	Cytoplasm	enzyme	
GAS6	GAS6	growth arrest specific 6	Extracellular Space	growth factor	
GCH1	GCH1	GTP cyclohydrolase 1	Cytoplasm	enzyme	
GDF15	GDF15	growth differentiation factor 15	Extracellular Space	growth factor	
GDNF	GDNF	glial cell derived	Extracellular Space	growth factor	

		neurotrophic factor			
GFAP	GFAP	glial fibrillary acidic protein	Cytoplasm	other	
GGT1	GGT1	gamma-glutamyltransferase 1	Plasma Membrane	enzyme	
GH1	GH1	growth hormone 1	Extracellular Space	growth factor	
GLUD1	GLUD1	glutamate dehydrogenase 1	Cytoplasm	enzyme	
GPT	GPT	glutamic--pyruvic transaminase	Cytoplasm	enzyme	
GPX1	GPX1	glutathione peroxidase 1	Cytoplasm	enzyme	
GSTA1	GSTA1	glutathione S-transferase alpha 1	Cytoplasm	enzyme	
GSTM1	GSTM1	glutathione S-transferase mu 1	Cytoplasm	enzyme	
GSTP1	GSTP1	glutathione S-transferase pi 1	Cytoplasm	enzyme	
GUSB	GUSB	glucuronidase beta	Cytoplasm	enzyme	
GZMA	GZMA	granzyme A	Cytoplasm	peptidase	
GZMB	GZMB	granzyme B	Cytoplasm	peptidase	
HAVCR2	HAVCR2	hepatitis A virus cellular receptor 2	Plasma Membrane	other	MBG453, LY3321367
HBA1	HBA1/HBA2	hemoglobin subunit alpha 2	Extracellular Space	transporter	iron dextran, mefloquine
HELLS	HELLS	helicase, lymphoid specific	Nucleus	enzyme	
HGF	HGF	hepatocyte growth factor	Extracellular Space	growth factor	rilotumumab, MP0250

HIF1A	HIF1A	hypoxia inducible factor 1 alpha subunit	Nucleus	transcription regulator	EZN 2968
HLA-A	HLA-A	major histocompatibility complex, class I, A	Plasma Membrane	other	
HLA-B	HLA-B	major histocompatibility complex, class I, B	Plasma Membrane	transmembrane receptor	
HLA-C	HLA-C	major histocompatibility complex, class I, C	Plasma Membrane	other	
HLA-DMA	HLA-DMA	major histocompatibility complex, class II, DM alpha	Plasma Membrane	transmembrane receptor	
HLA-DPB1	HLA-DPB1	major histocompatibility complex, class II, DP beta 1	Plasma Membrane	transmembrane receptor	
HLA-DQA1	HLA-DQA1	major histocompatibility complex, class II, DQ alpha 1	Plasma Membrane	transmembrane receptor	
HLA-DQB1	HLA-DQB1	major histocompatibility complex, class II, DQ beta 1	Plasma Membrane	other	

HLA-DRA	HLA-DRA	major histocompatibility complex, class II, DR alpha	Plasma Membrane	transmembrane receptor	
HLA-DRB1	HLA-DRB1	major histocompatibility complex, class II, DR beta 1	Plasma Membrane	transmembrane receptor	apolizumab
HLA-E	HLA-E	major histocompatibility complex, class I, E	Plasma Membrane	transmembrane receptor	
HLA-G	HLA-G	major histocompatibility complex, class I, G	Plasma Membrane	other	
HMGB1	HMGB1	high mobility group box 1	Nucleus	transcription regulator	
HMGCR	HMGCR	3-hydroxy-3-methylglutaryl-CoA reductase	Cytoplasm	enzyme	aspirin/pravastatin, beta-hydroxy simvastatin acid, ezetimibe/fluvastatin, atorvastatin/niacin, fenofibric acid/rosuvastatin, atorvastatin/ezetimibe, ezetimibe/rosuvastatin, simvastatin/sitagliptin, atorvastatin/choline fenofibrate, choline fenofibrate/simvastatin, fenofibrate/simvastatin, pitavastatin, lovastatin/niacin, ezetimibe/simvastatin, amlodipine/atorvastatin, fluvastatin, cerivastatin,

					atorvastatin, pravastatin, simvastatin, lovastatin, rosuvastatin
HMMR	HMMR	hyaluronan mediated motility receptor	Plasma Membrane	transmembrane receptor	
HMOX1	HMOX1	heme oxygenase 1	Cytoplasm	enzyme	tin mesoporphyrin
HP	HP	haptoglobin	Extracellular Space	peptidase	
HRH2	HRH2	histamine receptor H2	Plasma Membrane	G-protein coupled receptor	asenapine, famotidine/ibuprofen, cimetidine, famotidine, tesmilifene, triprolidine, doxepin, ranitidine, methantheline, metiamide, epinastine, nizatidine, buclizine
HSP90AA1	HSP90AA1	heat shock protein 90 alpha family class A member 1	Cytoplasm	enzyme	alvespimycin, retaspimycin, luminespib, TAS-116, cisplatin
HSPA1A	HSPA1A/HSPA1B	heat shock protein family A (Hsp70) member 1A	Cytoplasm	enzyme	
HSPA1L	HSPA1L	heat shock protein family A (Hsp70) member 1 like	Cytoplasm	other	
HSPA4	HSPA4	heat shock protein family A (Hsp70) member 4	Cytoplasm	other	
HSPB1	HSPB1	heat shock protein family B (small)	Cytoplasm	other	

		member 1			
HSPD1	HSPD1	heat shock protein family D (Hsp60) member 1	Cytoplasm	enzyme	
ICAM1	ICAM1	intercellular adhesion molecule 1	Plasma Membrane	transmembrane receptor	
ICOS	ICOS	inducible T-cell costimulator	Plasma Membrane	transmembrane receptor	
IDO1	IDO1	indoleamine 2,3-dioxygenase 1	Cytoplasm	enzyme	1-methyl-D-tryptophan, epacadostat, NLG919, KHK2455, PF-06840003
IFIH1	IFIH1	interferon induced with helicase C domain 1	Nucleus	enzyme	
IFNA2	IFNA2	interferon alpha 2	Extracellular Space	cytokine	
IFNG	IFNG	interferon gamma	Extracellular Space	cytokine	
IFNGR2	IFNGR2	interferon gamma receptor 2	Plasma Membrane	transmembrane receptor	interferon gamma-1b
IL10	IL10	interleukin 10	Extracellular Space	cytokine	
IL10RA	IL10RA	interleukin 10 receptor subunit alpha	Plasma Membrane	transmembrane receptor	
IL10RB	IL10RB	interleukin 10 receptor subunit beta	Plasma Membrane	transmembrane receptor	
IL11	IL11	interleukin 11	Extracellular Space	cytokine	
IL13	IL13	interleukin 13	Extracellular Space	cytokine	tralokinumab
IL15	IL15	interleukin 15	Extracellular Space	cytokine	
IL16	IL16	interleukin 16	Extracellular Space	cytokine	
IL17A	IL17A	interleukin	Extracellular	cytokine	secukinumab,

		17A	Space		ixekizumab, CJM112, bimekizumab
IL17F	IL17F	interleukin 17F	Extracellular Space	cytokine	bimekizumab
IL17RA	IL17RA	interleukin 17 receptor A	Plasma Membrane	transmembrane receptor	brodalumab
IL18	IL18	interleukin 18	Extracellular Space	cytokine	
IL18R1	IL18R1	interleukin 18 receptor 1	Plasma Membrane	transmembrane receptor	
IL1A	IL1A	interleukin 1 alpha	Extracellular Space	cytokine	
IL1B	IL1B	interleukin 1 beta	Extracellular Space	cytokine	canakinumab, gevokizumab, canakinumab/INS, gallium nitrate
IL1R1	IL1R1	interleukin 1 receptor type 1	Plasma Membrane	transmembrane receptor	anakinra
IL1RL1	IL1RL1	interleukin 1 receptor like 1	Plasma Membrane	transmembrane receptor	
IL1RN	IL1RN	interleukin 1 receptor antagonist	Extracellular Space	cytokine	
IL2	IL2	interleukin 2	Extracellular Space	cytokine	
IL21	IL21	interleukin 21	Extracellular Space	cytokine	
IL22	IL22	interleukin 22	Extracellular Space	cytokine	
IL23A	IL23A	interleukin 23 subunit alpha	Extracellular Space	cytokine	LY3074828
IL23R	IL23R	interleukin 23 receptor	Plasma Membrane	transmembrane receptor	
IL2RA	IL2RA	interleukin 2 receptor subunit alpha	Plasma Membrane	transmembrane receptor	LMB-2, daclizumab, basiliximab, aldesleukin, denileukin diftitox
IL2RB	IL2RB	interleukin 2	Plasma	transmemb	monoclonal antibody

		receptor subunit beta	Membrane	rane receptor	Mik-Beta-1, daclizumab, aldesleukin, denileukin diftitox
IL2RG	IL2RG	interleukin 2 receptor subunit gamma	Plasma Membrane	transmembrane receptor	aldesleukin, denileukin diftitox
IL3	IL3	interleukin 3	Extracellular Space	cytokine	
IL3RA	IL3RA	interleukin 3 receptor subunit alpha	Plasma Membrane	transmembrane receptor	sargramostim, EPO/sargramostim, rituximab/sargramostim, DT388IL3, talacotuzumab
IL4	IL4	interleukin 4	Extracellular Space	cytokine	
IL4R	IL4R	interleukin 4 receptor	Plasma Membrane	transmembrane receptor	
IL5	IL5	interleukin 5	Extracellular Space	cytokine	mepolizumab, reslizumab
IL6	IL6	interleukin 6	Extracellular Space	cytokine	tocilizumab, siltuximab
IL6R	IL6R	interleukin 6 receptor	Plasma Membrane	transmembrane receptor	tocilizumab, sarilumab, methotrexate/tocilizumab, methotrexate/sarilumab
IL7	IL7	interleukin 7	Extracellular Space	cytokine	
IL7R	IL7R	interleukin 7 receptor	Plasma Membrane	transmembrane receptor	recombinant human interleukin-7
IL9	IL9	interleukin 9	Extracellular Space	cytokine	
INS	INS	insulin	Extracellular Space	other	
IRF1	IRF1	interferon regulatory factor 1	Nucleus	transcription regulator	
ITGA5	ITGA5	integrin subunit alpha 5	Plasma Membrane	transmembrane receptor	
ITGAL	ITGAL	integrin subunit alpha L	Plasma Membrane	transmembrane receptor	efalizumab
ITGAM	ITGAM	integrin	Plasma	transmemb	

		subunit alpha M	Membrane	rane receptor	
ITGB1	ITGB1	integrin subunit beta 1	Plasma Membrane	transmembrane receptor	
ITGB2	ITGB2	integrin subunit beta 2	Plasma Membrane	transmembrane receptor	
ITGB3	ITGB3	integrin subunit beta 3	Plasma Membrane	transmembrane receptor	abciximab, TP 9201, cilengitide, tirofiban
ITGB7	ITGB7	integrin subunit beta 7	Plasma Membrane	transmembrane receptor	vedolizumab
KCNJ5	KCNJ5	potassium voltage-gated channel subfamily J member 5	Plasma Membrane	ion channel	minoxidil, nicorandil, amiodarone
KDR	KDR	kinase insert domain receptor	Plasma Membrane	kinase	AEE 788, sunitinib, cediranib, pazopanib, axitinib, tesevatinib, CEP 7055, brivanib alaninate, CHIR-265, lenvatinib, tivozanib, BMS-690514, motesanib, OSI-930, telatinib, XL999, brivanib, ramucirumab, cabozantinib, nintedanib, XL820, pegdinetanib, foretinib, regorafenib, golvatinib, TAK 593, apatinib, CP-547632, PF 00337210, JI 101, CYC 116, bortezomib/sorafenib, lapatinib/pazopanib, dexamethasone/lenalidomide/sorafenib, bevacizumab/sorafenib, rebastinib, MGCD-265, cabozantinib/erlotinib, famitinib, lucitanib, paclitaxel/ramucirumab,

					VEGF inhibitor drug, docetaxel/ramucirumab, cytarabine/idarubicin/sorafenib, 5-azacytidine/sorafenib, decitabine/sorafenib, fruquintinib, altiratinib, MGCD516, anlotinib, SAR103168, pexmetinib, sulfatinib, VEGFR2-targeted Contrast Agent BR55, everolimus/sorafenib, docetaxel/nintedanib, everolimus/lenvatinib, VEGFR2 inhibitor, vatalanib, sorafenib, sorafenib/sulindac/sunitinib, vandetanib, pegaptanib, semaxinib, orantinib, midostaurin
KITLG	KITLG	KIT ligand	Extracellular Space	growth factor	
KLRK1	KLRC4-KLRK1/KLRK1	killer cell lectin like receptor K1	Plasma Membrane	transmembrane receptor	
KLRD1	KLRD1	killer cell lectin like receptor D1	Plasma Membrane	transmembrane receptor	
KNG1	KNG1	kininogen 1	Extracellular Space	other	
KRT10	KRT10	keratin 10	Cytoplasm	other	
KRT19	KRT19	keratin 19	Cytoplasm	other	
KRT8	KRT8	keratin 8	Cytoplasm	other	
LBP	LBP	lipopolysaccharide binding protein	Plasma Membrane	transporter	
LCN2	LCN2	lipocalin 2	Extracellular Space	transporter	
LEP	LEP	leptin	Extracellular Space	growth factor	
LIF	LIF	LIF, interleukin 6	Extracellular Space	cytokine	

		family cytokine			
LIG3	LIG3	DNA ligase 3	Nucleus	enzyme	
LPA	LPA	lipoprotein(a)	Extracellular Space	other	
LPL	LPL	lipoprotein lipase	Cytoplasm	enzyme	atorvastatin/niacin, nicotinic acid/pioglitazone, nicotinic acid, tyloxapol, lovastatin/niacin
LTA	LTA	lymphotoxin alpha	Extracellular Space	cytokine	etanercept, etanercept/methotrexate
LTF	LTF	lactotransferrin	Extracellular Space	peptidase	
LY96	LY96	lymphocyte antigen 96	Plasma Membrane	transmembrane receptor	
MAPK1	MAPK1	mitogen-activated protein kinase 1	Cytoplasm	kinase	MAP kinase1 inhibitor, binimetinib, ulixertinib, LY-3007113, pexmetinib, LY3214996, LTT462
MAPK14	MAPK14	mitogen-activated protein kinase 14	Cytoplasm	kinase	talmapimod, RO-3201195, ralimetinib, p38 MAP kinase inhibitor
MAPK8	MAPK8	mitogen-activated protein kinase 8	Cytoplasm	kinase	aplidine
MB	MB	myoglobin	Cytoplasm	transporter	
MIF	MIF	macrophage migration inhibitory factor	Extracellular Space	cytokine	
MKI67	MKI67	marker of proliferation Ki-67	Nucleus	other	
MMP1	MMP1	matrix metallopeptidase 1	Extracellular Space	peptidase	rebimastat, marimastat
MMP14	MMP14	matrix metallopeptidase 14	Extracellular Space	peptidase	rebimastat, marimastat, prinomastat
MMP19	MMP19	matrix metallopepti	Extracellular Space	peptidase	marimastat

		dase 19			
MMP2	MMP2	matrix metallopeptidase 2	Extracellular Space	peptidase	MMP2 MMP9 inhibitor, rebimastat, marimastat, prinomastat
MMP3	MMP3	matrix metallopeptidase 3	Extracellular Space	peptidase	marimastat
MMP7	MMP7	matrix metallopeptidase 7	Extracellular Space	peptidase	marimastat
MMP8	MMP8	matrix metallopeptidase 8	Extracellular Space	peptidase	rebimastat, marimastat
MMP9	MMP9	matrix metallopeptidase 9	Extracellular Space	peptidase	MMP2 MMP9 inhibitor, GS-5745, rebimastat, marimastat, prinomastat, glucosamine
MPO	MPO	myeloperoxidase	Cytoplasm	enzyme	
MTHFR	MTHFR	methylenetetrahydrofolate reductase	Cytoplasm	enzyme	
MUC1	MUC1	mucin 1, cell surface associated	Plasma Membrane	other	HuHMFG1, yttrium y 90 clivatuzumab tetraxetan, GO-203-2C
MUTYH	MUTYH	mutY DNA glycosylase	Nucleus	enzyme	
MX1	MX1	MX dynamin like GTPase 1	Cytoplasm	enzyme	
MYC	MYC	MYC proto-oncogene, bHLH transcription factor	Nucleus	transcription regulator	
MYD88	MYD88	myeloid differentiation primary response 88	Plasma Membrane	other	IMO-8400
MYH9	MYH9	myosin heavy chain 9	Cytoplasm	enzyme	
C5	NADPH		Other	chemical - endogenou	

				s mammalian	
NCAM1	NCAM1	neural cell adhesion molecule 1	Plasma Membrane	other	BB-10901
NEFL	NEFL	neurofilament light	Cytoplasm	other	
NFE2L2	NFE2L2	nuclear factor, erythroid 2 like 2	Nucleus	transcription regulator	RTA 408
NGF	NGF	nerve growth factor	Extracellular Space	growth factor	
NLRP3	NLRP3	NLR family pyrin domain containing 3	Cytoplasm	other	
NOD2	NOD2	nucleotide binding oligomerization domain containing 2	Cytoplasm	other	
NOS2	NOS2	nitric oxide synthase 2	Cytoplasm	enzyme	pimagedine, triflusal, GW 273629, targinine
NOS3	NOS3	nitric oxide synthase 3	Cytoplasm	enzyme	5,6,7,8-tetrahydrobiopterin, GW 273629, targinine
OGG1	OGG1	8-oxoguanine DNA glycosylase	Nucleus	enzyme	
PADI4	PADI4	peptidyl arginine deiminase 4	Cytoplasm	enzyme	
PARP1	PARP1	poly(ADP-ribose) polymerase 1	Nucleus	enzyme	poly ADP ribose polymerase 1 inhibitor, veliparib, rucaparib, olaparib, niraparib, E7449, ABT-767, CEP-9722, fluzoparib, SC10914, simmiparib, INO-1001
PCNA	PCNA	proliferating cell nuclear	Nucleus	enzyme	

		antigen			
PDCD1	PDCD1	programmed cell death 1	Plasma Membrane	phosphatase	pidilizumab, nivolumab, pembrolizumab, ipilimumab/nivolumab, anti PD-1 antibody, SHR-1210, JS001, TSR-042, PF-06801591, JNJ-63723283, BI 754091, AGEN2034
PDGFB	PDGFB	platelet derived growth factor subunit B	Extracellular Space	growth factor	
PDGFRA	PDGFRA	platelet derived growth factor receptor alpha	Plasma Membrane	kinase	dasatinib, sunitinib, pazopanib, axitinib, lenvatinib, telatinib, amuvatinib, nintedanib, regorafenib, olaratumab, lapatinib/pazopanib, imatinib/sirolimus, crenolanib, DCC-2618, BLU-285, docetaxel/nintedanib, doxorubicin/olaratumab, everolimus/lenvatinib, imatinib, midostaurin, becaplermin
PDGFRB	PDGFRB	platelet derived growth factor receptor beta	Plasma Membrane	kinase	dasatinib, sunitinib, pazopanib, axitinib, tivozanib, telatinib, tandutinib, nintedanib, regorafenib, JI 101, bortezomib/sorafenib, lapatinib/pazopanib, dexamethasone/lenalidomide/sorafenib, bevacizumab/sorafenib, imatinib/sirolimus, cytarabine/idarubicin/sorafenib, 5-azacytidine/sorafenib, decitabine/sorafenib, everolimus/sorafenib,

					docetaxel/nintedanib, imatinib, sorafenib, sorafenib/sulindac/sunitinib, orantinib, midostaurin, becaplermin
PECAM1	PECAM1	platelet and endothelial cell adhesion molecule 1	Plasma Membrane	other	
PF4	PF4	platelet factor 4	Extracellular Space	cytokine	
PGR	PGR	progesterone receptor	Nucleus	ligand-dependent nuclear receptor	misoprostol, dienogest, ulipristal acetate, asoprisnil, ethynodiol diacetate, norethindrone acetate, ethinyl estradiol/ethynodiol diacetate, estradiol cypionate/medroxyprogesterone acetate, tosagestin, estradiol/norethindrone acetate, ZK 230211, etonogestrel, nestorone, telapristone acetate, ethinyl estradiol/etonogestrel, 17alpha-hydroxyprogesterone caproate, leuprolide acetate/norethindrone acetate, megestrol acetate/tamoxifen, ulipristal, tanaproget, progesterone receptor antagonist, fluorine F 18 fluoro furanyl norprogesterone, desogestrel/ethinyl estradiol, drospirenone/ethinyl estradiol, ethinyl estradiol/norelgestromin, ethinyl estradiol/norethindrone, ethinyl

					estradiol/levonorgestrel, ethinyl estradiol/norgestrel, ethinyl estradiol/norgestimate, mometasone furoate, megestrol acetate, onapristone, drospirenone, medroxyprogesterone acetate, norgestrel, dydrogesterone, desogestrel, danazol, levonorgestrel, norelgestromin, mifepristone, norethindrone, norgestimate, progesterone
PI3	PI3	peptidase inhibitor 3	Extracellular Space	other	
PIK3C2A	PIK3C2A	phosphatidylinositol-4-phosphate 3-kinase catalytic subunit type 2 alpha	Cytoplasm	kinase	
PIK3CG	PIK3CG	phosphatidylinositol-4,5-bisphosphate 3-kinase catalytic subunit gamma	Cytoplasm	kinase	SF 1126, PX-866, PI3Kg inhibitor, dactolisib, pictilisib, buparlisib, XL147, PQR309, RP6530
PLAT	PLAT	plasminogen activator, tissue type	Extracellular Space	peptidase	6-aminocaproic acid
PLAU	PLAU	plasminogen activator, urokinase	Extracellular Space	peptidase	
PLAUR	PLAUR	plasminogen activator, urokinase receptor	Plasma Membrane	transmembrane receptor	

PML	PML	promyelocytic leukemia	Nucleus	transcription regulator	arsenic trioxide
PNP	PNP	purine nucleoside phosphorylase	Nucleus	enzyme	forodesine, PD 141955, purine nucleoside phosphorylase inhibitor
POLB	POLB	DNA polymerase beta	Nucleus	enzyme	nelarabine, cytarabine/fludarabine phosphate, lamivudine/nelfinavir/stavudine, cytarabine/daunorubicin, lamivudine/stavudine, lamivudine/nevirapine/stavudine, cladribine/cytarabine/daunorubicin, cytarabine/daunorubicin/tretinoin, cytarabine/idarubicin, cytarabine/etoposide, cytarabine/etoposide/mitoxantrone, cytarabine/dexamethasone, 5-azacytidine/cytarabine/decitabine, cytarabine/mitoxantrone, cytarabine/methotrexate, cladribine/cytarabine/filgrastim, clofarabine/cyclophosphamide/etoposide, cytarabine/filgrastim/fludarabine phosphate, cladribine/cytarabine/filgrastim/mitoxantrone, clofarabine/cytarabine/filgrastim, cytarabine/filgrastim/fludarabine phosphate/idarubicin, cladribine/cytarabine/filgrastim/idarubicin,

					cytarabine/dexamethasone/methotrexate, cladribine/cytarabine/filgrastim/idarubicin/plerixafor, cytarabine/doxorubicin, clofarabine/cytarabine/filgrastim/idarubicin, clofarabine/filgrastim, cytarabine/topotecan, cytarabine/idarubicin/sorafenib, cladribine/cytarabine/decitabine, cytarabine/rituximab, clofarabine, cytarabine, stavudine, trifluridine, vidarabine, zalcitabine, entecavir
POMC	POMC	proopiomelanocortin	Extracellular Space	other	
PON1	PON1	paraoxonase 1	Extracellular Space	phosphatase	
PPARG	PPARG	peroxisome proliferator activated receptor gamma	Nucleus	ligand-dependent nuclear receptor	icosapent, amlodipine/telmisartan, inolitazone, aleglitazar, aspirin/dipyridamole/telmisartan, clopidogrel/telmisartan, glimepiride/rosiglitazone, nicotinic acid/pioglitazone, alogliptin/pioglitazone, glimepiride/pioglitazone, GED-0507-34-levo, INS/pioglitazone, rosiglitazone, farglitazar, sulfasalazine, pioglitazone, telmisartan, tesaglitazar, troglitazone, hydrochlorothiazide/telmisartan, balsalazide, mesalamine, bezafibrate
PPARGC1A	PPARGC1A	PPARG coactivator 1	Nucleus	transcription regulator	

		alpha			
PPBP	PPBP	pro-platelet basic protein	Extracellular Space	cytokine	
PRF1	PRF1	perforin 1	Cytoplasm	transporter	
PRL	PRL	prolactin	Extracellular Space	cytokine	
PRTN3	PRTN3	proteinase 3	Extracellular Space	peptidase	
PTEN	PTEN	phosphatase and tensin homolog	Cytoplasm	phosphatase	
PTGS2	PTGS2	prostaglandin-endoperoxide synthase 2	Cytoplasm	enzyme	acetaminophen/pentazocine, acetaminophen/clemastine/pseudoephedrine, aspirin/butalbital/caffeine, acetaminophen/caffeine/dihydrocodeine, aspirin/hydrocodone, aspirin/oxycodone, acetaminophen/aspirin/caffeine, aspirin/pravastatin, acetaminophen/dexbrompheniramine/pseudoephedrine, aspirin/meprobamate, aspirin/caffeine/propoxyphene, aspirin/butalbital/caffeine/codeine, aspirin/caffeine/dihydrocodeine, chlorpheniramine/ibuprofen/pseudoephedrine, licofelone, menatetrenone, icosapent, suprofen, lornoxicam, tiaprofenic acid, lumiracoxib, tenoxicam, naproxen/sumatriptan, apricoxib, parecoxib, ibuprofen/phenylephrine, acetaminophen/aspirin/codeine, esomeprazole/naproxen,

					aspirin/esomeprazole, aspirin/dipyridamole/telmisartan, famotidine/ibuprofen, aspirin/dabigatran etexilate, diclofenac/omeprazole, chlorpheniramine/ibuprofen/phenylephrine, dexamethasone/pomalidomide, sulindac/tamoxifen, sulindac/toremifene, raloxifene/sulindac, ketorolac/phenylephrine, aspirin/bivalirudin, diclofenac/hyaluronic acid, aspirin/clopidogrel, aspirin/omeprazole, aspirin/enoxaparin, aspirin/lisinopril, COX2 inhibitor, diclofenac/misoprostol, acetaminophen/butalbital/caffeine, hydrocodone/ibuprofen, acetaminophen/hydrocodone, acetaminophen/tramadol, acetaminophen/codeine, acetaminophen/oxycodone, acetaminophen/propoxyphene, niflumic acid, nitroaspirin, ketoprofen, diclofenac, etoricoxib, naproxen, meclofenamic acid, pomalidomide, meloxicam, celecoxib, ibuprofen/pseudoephedrine, diphenhydramine/ibuprofen, dipyrone, nimesulide, acetaminophen, mefenamic acid,

					bortezomib/dexamethasone/pomalidomide, diflunisal, ibuprofen, GW406381X, phenylbutazone, indomethacin, sulfasalazine, piroxicam, valdecoxib, aspirin, carprofen, zomepirac, rofecoxib, sorafenib/sulindac/sunitinib, aspirin/caffeine/orphenadrine, acetaminophen/butalbital, balsalazide, aspirin/dipyridamole, acetaminophen/butalbital/caffeine/codeine, racemic flurbiprofen, phenacetin, sulindac, nabumetone, etodolac, tolmetin, ketorolac, oxaprozin, mesalamine, salsalate, fenoprofen, salicylic acid, acetaminophen/caffeine/chlorpheniramine/hydrocodone/phenylephrine, bromfenac
PTPN22	PTPN22	protein tyrosine phosphatase, non-receptor type 22	Cytoplasm	phosphatase	
RAC1	RAC1	Rac family small GTPase 1	Plasma Membrane	enzyme	
RBP4	RBP4	retinol binding protein 4	Extracellular Space	transporter	
RHOA	RHOA	ras homolog family member A	Cytoplasm	enzyme	
RPL5	RPL5	ribosomal	Cytoplasm	other	

		protein L5			
S100A8	S100A8	S100 calcium binding protein A8	Cytoplasm	other	
S100A9	S100A9	S100 calcium binding protein A9	Cytoplasm	other	
S100B	S100B	S100 calcium binding protein B	Cytoplasm	other	
SCGB1A1	SCGB1A1	secretoglobin family 1A member 1	Extracellular Space	cytokine	
SELE	SELE	selectin E	Plasma Membrane	transmembrane receptor	
SELL	SELL	selectin L	Plasma Membrane	transmembrane receptor	
SELP	SELP	selectin P	Plasma Membrane	transmembrane receptor	
SERPINA1	SERPINA1	serpin family A member 1	Extracellular Space	other	
SERPINA3	SERPINA3	serpin family A member 3	Extracellular Space	other	
SERPINC1	SERPINC1	serpin family C member 1	Extracellular Space	enzyme	dalteparin, heparin, enoxaparin, ardeparin, SR-123781A, low molecular weight heparin, glucuronyl glucosamine glycan sulfate, semuloparin, aspirin/enoxaparin, fondaparinux, nadroparin
SERPINE1	SERPINE1	serpin family E member 1	Extracellular Space	other	drotrecogin alfa
SERPIN	SERPING	serpin	Extracellular	other	

G1	1	family G member 1	Space		
SETD2	SETD2	SET domain containing 2	Cytoplasm	enzyme	
SLC17A5	SLC17A5	solute carrier family 17 member 5	Plasma Membrane	transporter	
SLPI	SLPI	secretory leukocyte peptidase inhibitor	Cytoplasm	other	
SMUG1	SMUG1	single-strand-selective monofunctional uracil-DNA glycosylase 1	Nucleus	enzyme	
SOCS3	SOCS3	suppressor of cytokine signaling 3	Cytoplasm	phosphatase	
SOD1	SOD1	superoxide dismutase 1	Cytoplasm	enzyme	
SOD2	SOD2	superoxide dismutase 2	Cytoplasm	enzyme	
SOD3	SOD3	superoxide dismutase 3	Extracellular Space	enzyme	
SPP1	SPP1	secreted phosphoprotein 1	Extracellular Space	cytokine	
SST	SST	somatostatin	Extracellular Space	other	
STAT1	STAT1	signal transducer and activator of transcription 1	Nucleus	transcription regulator	
STAT3	STAT3	signal transducer and activator of transcription 3	Nucleus	transcription regulator	OPB-31121, OPB-51602

STAT4	STAT4	signal transducer and activator of transcription 4	Nucleus	transcription regulator	
STAT5A	STAT5A	signal transducer and activator of transcription 5A	Nucleus	transcription regulator	
STAT5B	STAT5B	signal transducer and activator of transcription 5B	Nucleus	transcription regulator	
STAT6	STAT6	signal transducer and activator of transcription 6	Nucleus	transcription regulator	
SYP	SYP	synaptophysin	Cytoplasm	transporter	
TAP1	TAP1	transporter 1, ATP binding cassette subfamily B member	Cytoplasm	transporter	
TBX21	TBX21	T-box 21	Nucleus	transcription regulator	
TCIRG1	TCIRG1	T-cell immune regulator 1, ATPase H+ transporting V0 subunit a3	Plasma Membrane	enzyme	
TFRC	TFRC	transferrin receptor	Plasma Membrane	transporter	CALAA-01
TGFA	TGFA	transforming	Extracellular	growth	

		growth factor alpha	Space	factor	
TGFB1	TGFB1	transforming growth factor beta 1	Extracellular Space	growth factor	dalantercept, LY3200882
TGFB2	TGFB2	transforming growth factor beta 2	Extracellular Space	growth factor	trabedersen, dalantercept
TGFB3	TGFB3	transforming growth factor beta 3	Extracellular Space	growth factor	
TGFBR2	TGFBR2	transforming growth factor beta receptor 2	Plasma Membrane	kinase	
TGIF1	TGIF1	TGFB induced factor homeobox 1	Nucleus	transcription regulator	
TH	TH	tyrosine hydroxylase	Cytoplasm	enzyme	tyrosine hydroxylase inhibitor, 5,6,7,8-tetrahydrobiopterin, metyrosine, alpha-methylparatyrosine
THBD	THBD	thrombomodulin	Plasma Membrane	transmembrane receptor	
THBS1	THBS1	thrombospondin 1	Extracellular Space	other	
THPO	THPO	thrombopoietin	Extracellular Space	cytokine	
TIMP1	TIMP1	TIMP metallopeptidase inhibitor 1	Extracellular Space	cytokine	
TIMP3	TIMP3	TIMP metallopeptidase inhibitor 3	Extracellular Space	other	
TKT	TKT	transketolase	Cytoplasm	enzyme	
TLR1	TLR1	toll like receptor 1	Plasma Membrane	transmembrane receptor	
TLR10	TLR10	toll like	Plasma	transmemb	

		receptor 10	Membrane	rane receptor	
TLR2	TLR2	toll like receptor 2	Plasma Membrane	transmembrane receptor	OM 174 lipid
TLR3	TLR3	toll like receptor 3	Plasma Membrane	transmembrane receptor	rintatolimod
TLR4	TLR4	toll like receptor 4	Plasma Membrane	transmembrane receptor	resatorvid, OM 174 lipid
TLR5	TLR5	toll like receptor 5	Plasma Membrane	transmembrane receptor	
TLR6	TLR6	toll like receptor 6	Plasma Membrane	transmembrane receptor	
TLR9	TLR9	toll like receptor 9	Plasma Membrane	transmembrane receptor	agatolimod, HYB-2055, GNKG168, SD-101, MGN1703, hydroxychloroquine
TNC	TNC	tenascin C	Extracellular Space	other	
TNF	TNF	tumor necrosis factor	Extracellular Space	cytokine	adalimumab, etanercept, infliximab, certolizumab, golimumab, tumor necrosis factor receptor antagonist, infliximab/methotrexate, dexamethasone/thalidomide, dexamethasone/pomalidomide, cyclophosphamide/dexamethasone/thalidomide, golimumab/methotrexate, bortezomib/dexamethasone/thalidomide, rituximab/thalidomide, bortezomib/thalidomide, prednisone/thalidomide, adalimumab/methotrexate, etanercept/methotrexate, pomalidomide,

					bortezomib/dexamethasone/pomalidomide, thalidomide
TNFRSF10B	TNFRSF10B	TNF receptor superfamily member 10b	Plasma Membrane	transmembrane receptor	tigatuzumab, conatumumab, DS-8273a, RO6874813
TNFRSF11B	TNFRSF11B	TNF receptor superfamily member 11b	Plasma Membrane	transmembrane receptor	
TNFRSF13C	TNFRSF13C	TNF receptor superfamily member 13C	Plasma Membrane	transmembrane receptor	
TNFRSF18	TNFRSF18	TNF receptor superfamily member 18	Plasma Membrane	transmembrane receptor	MK-1248, INCAGN01876
TNFRSF1A	TNFRSF1A	TNF receptor superfamily member 1A	Plasma Membrane	transmembrane receptor	GSK2862277
TNFRSF1B	TNFRSF1B	TNF receptor superfamily member 1B	Plasma Membrane	transmembrane receptor	
TNFRSF4	TNFRSF4	TNF receptor superfamily member 4	Plasma Membrane	transmembrane receptor	GSK3174998, PF-04518600, MOXR0916, MEDI0562
TNFRSF8	TNFRSF8	TNF receptor superfamily member 8	Plasma Membrane	transmembrane receptor	HeFi-1 monoclonal antibody, brentuximab vedotin, brentuximab vedotin/rituximab, AFM13
TNFRSF9	TNFRSF9	TNF receptor superfamily member 9	Plasma Membrane	transmembrane receptor	urelumab
TNFSF10	TNFSF10	TNF superfamily member 10	Extracellular Space	cytokine	
TNFSF1	TNFSF11	TNF	Extracellular	cytokine	lenalidomide, denosumab,

1		superfamily member 11	Space		lenalidomide/temsirolimus, dexamethasone/lenalidomide/sorafenib, dexamethasone/lenalidomide, bortezomib/dexamethasone/lenalidomide, carfilzomib/dexamethasone/lenalidomide, lenalidomide/rituximab, denosumab/levothyroxine, erythropoietin/lenalidomide, bortezomib/lenalidomide, TK006
TNFSF13B	TNFSF13B	TNF superfamily member 13b	Extracellular Space	cytokine	belimumab
TNNI3	TNNI3	troponin I3, cardiac type	Cytoplasm	transporter	
TP53	TP53	tumor protein p53	Nucleus	transcription regulator	APR-246, cenersen, CGM097, kevetrin, azurin 50-77, COTI-2
TP63	TP63	tumor protein p63	Nucleus	transcription regulator	
TTR	TTR	transthyretin	Extracellular Space	transporter	4'-iodo-4'-deoxydoxorubicin
TUBB3	TUBB3	tubulin beta 3 class III	Cytoplasm	other	epothilone B, ixabepilone, colchicine/probenecid, larotaxel, ABT-751, eribulin, simotaxel, davunetide, vintafolide, milataxel, cevipabulin, gemcitabine/paclitaxel, docetaxel/prednisone, capecitabine/docetaxel, paclitaxel/trastuzumab, capecitabine/ixabepilone, cyclophosphamide/prednisone/vincristine, docetaxel/hydrocortisone, cyclophosphamide/docetaxel,

					gemcitabine/vinorelbine, cyclophosphamide/daunorubicin/imatinib/prednisone/vincristine, cyclophosphamide/topotecan/vincristine, docetaxel/gemcitabine, docetaxel/gemcitabine/vincristine, irinotecan/vincristine, irinotecan/temozolomide/vincristine, bevacizumab/paclitaxel, cyclophosphamide/docetaxel/epirubicin/5-fluorouracil/trastuzumab, docetaxel/trastuzumab, trastuzumab/vinorelbine, gemcitabine/oxaliplatin/paclitaxel, cyclophosphamide/epirubicin/vincristine, docetaxel/irinotecan, docetaxel/5-fluorouracil/oxaliplatin, capecitabine/docetaxel/gemcitabine, L-asparaginase/prednisone/vincristine, cyclophosphamide/etoposide/prednisone/rituximab/vincristine, cyclophosphamide/vinorelbine, cyclophosphamide/mitoxantrone/prednisone/vincristine, cyclophosphamide/etoposide/prednisone/vincristine, cyclophosphamide/prednisone/rituximab/vincristine, cyclophosphamide/mitoxantrone/prednisone/rituxim

					ab/vincristine, plinabulin, docetaxel/epirubicin, docetaxel/paclitaxel, epirubicin/paclitaxel, bevacizumab/paclitaxel/topotecan, paclitaxel/topotecan, bevacizumab/docetaxel, cyclophosphamide/prednisolone/vincristine, cyclophosphamide/prednisolone/rituximab/vincristine, cyclophosphamide/epirubicin/5-fluorouracil/vinorelbine, cyclophosphamide/epirubicin/5-fluorouracil/paclitaxel/trastuzumab, cyclophosphamide/epirubicin/5-fluorouracil/paclitaxel, cyclophosphamide/docetaxel/epirubicin/5-fluorouracil, cyclophosphamide/docetaxel/trastuzumab, cyclophosphamide/gemcitabine/prednisolone/rituximab/vincristine, cyclophosphamide/epirubicin/prednisone/vincristine, docetaxel/prednisolone, prednisone/vincristine, dexamethasone/vincristine, BMS-275183, eribulin/trastuzumab, dexamethasone/imatinib/vincristine, docetaxel, paclitaxel/rituximab, cyclophosphamide/epirubicin/prednisone/rituximab/vincristine, vinflunine, vinorelbine, vincristine,

					vinblastine, paclitaxel, podophyllotoxin, colchicine
TXN	TXN	thioredoxin	Cytoplasm	enzyme	PX-12
TYMS	TYMS	thymidylate synthetase	Nucleus	enzyme	flucytosine, 5-fluorouracil, tegafur, trimethoprim, cyclophosphamide/epirubicin/5-fluorouracil, pralatrexate, tegafur/uracil, 5-fluorouracil/oxaliplatin, capecitabine/irinotecan, 5-fluorouracil/irinotecan, capecitabine/docetaxel, capecitabine/lapatinib, capecitabine/ixabepilone, bevacizumab/5-fluorouracil, bevacizumab/capecitabine/oxaliplatin, cyclophosphamide/docetaxel/epirubicin/5-fluorouracil/trastuzumab, capecitabine/oxaliplatin, bevacizumab/pemetrexed, capecitabine/trastuzumab, capecitabine/gemcitabine, docetaxel/5-fluorouracil/oxaliplatin, epirubicin/5-fluorouracil/ oxaliplatin, capecitabine/docetaxel/gemcitabine, capecitabine/epirubicin/oxaliplatin, capecitabine/erlotinib, capecitabine/irinotecan/oxaliplatin, bevacizumab/capecitabine/irinotecan/oxaliplatin, bevacizumab/capecitabine, capecitabine/temozolomid

					e, 5-fluorouracil/irinotecan/oxaliplatin, leucovorin/methotrexate, 5-fluorouracil/gemcitabine, 5-fluorouracil/imiquimod, gimeracil/oxonic acid/tegafur, gemcitabine/pemetrexed, cyclophosphamide/epirubicin/5-fluorouracil/vinorelbine, cyclophosphamide/epirubicin/5-fluorouracil/paclitaxel/trastuzumab, cyclophosphamide/epirubicin/5-fluorouracil/paclitaxel, cyclophosphamide/docetaxel/epirubicin/5-fluorouracil, capecitabine/5-fluorouracil, capecitabine/5-fluorouracil/oxaliplatin, thymidylate synthase inhibitor, DFP-11207, BGC945, capecitabine/pertuzumab/trastuzumab, raltitrexed, plevitrexed, nolatrexed, 1843U89, leucovorin, capecitabine, trifluridine, floxuridine, pemetrexed
TYR	TYR	tyrosinase	Cytoplasm	enzyme	hydroquinone, azelaic acid
VCAM1	VCAM1	vascular cell adhesion molecule 1	Plasma Membrane	transmembrane receptor	
VDR	VDR	vitamin D receptor	Nucleus	transcription regulator	calcipotriene, ergocalciferol, ILX-23-7553, alendronate/cholecalciferol,

					2-(3-hydroxypropoxy)calcitriol, betamethasone dipropionate/calcipotriene, alfacalcidol, calcium carbonate/cholecalciferol, paricalcitol, doxercalciferol, cholecalciferol, calcitriol, calcifediol
VEGFA	VEGFA	vascular endothelial growth factor A	Extracellular Space	growth factor	dalteparin, bevacizumab, ranibizumab, aflibercept, bevacizumab/erlotinib, bevacizumab/sorafenib, bevacizumab/5-fluorouracil, bevacizumab/temozolomide, bevacizumab/irinotecan, bevacizumab/carmustine/lomustine, bevacizumab/paclitaxel, bevacizumab/irinotecan/oxaliplatin, aflibercept/irinotecan, bevacizumab/capecitabine/oxaliplatin, bevacizumab/panitumumab, bevacizumab/cetuximab, bevacizumab/pemetrexed, bevacizumab/gemcitabine, bevacizumab/capecitabine/irinotecan/oxaliplatin, bevacizumab/capecitabine, bevacizumab/paclitaxel/topotecan, bevacizumab/oxaliplatin, bevacizumab/docetaxel, bevacizumab-IRDye 800CW, bevacizumab/doxorubicin/paclitaxel,

					bevacizumab/IFNA2A, bevacizumab/topotecan, bevacizumab/doxorubicin, vanucizumab, pegaptanib
VIM	VIM	vimentin	Cytoplasm	other	
VWF	VWF	von Willebrand factor	Extracellular Space	other	
XDH	XDH	xanthine dehydrogenase	Cytoplasm	enzyme	allopurinol, oxypurinol, febuxostat, xanthine oxidase inhibitor, allopurinol/colchicine
XRCC1	XRCC1	X-ray repair cross complementing 1	Nucleus	enzyme	
MTOR	MTOR	mechanistic target of rapamycin kinase	Cytoplasm	enzyme	Rapamycin
RELA	RELA	RELA proto-oncogene	Cytoplasm /Nucleus	transcription factor	
