# Supplementary material for: Network Pharmacology-Guided Development of a Novel Integrative Regimen to Prevent Acute Graft-vs.-Host Disease
Source: Front Pharmacol. 2018 Dec 13;9:1440. doi: 10.3389/fphar.2018.01440 (PMC6300759; doi:10.3389/fphar.2018.01440)
Supplement: Supplementary file 5 [file Table_2.PDF]

Supplemental Table 2. Compounds in XBJ ingredients that regulate aGVHD genes						
Herb	Symbol	Synonym(s)	Family	PubChem ID	HMDB ID	CAS ID
3	protocatechualdehyde	3,4-dihydroxybenzaldehyde, 3,4-dihydroxybenzenecarbonal, 4-formyl-1,2-dihydroxybenzene, 139-85-5, benzaldehyde, 3,4-dihydroxy-, benzaldehyde, 3,4-dihydroxy-(9CI), C7H6O3, EINECS 205-377-7, NSC 22961, rancinamycin IV	chemical - endogenous non-mammalian	8768	HMDB0059965	139-85-5
1	3-n-butylphthalide	1(3H)-isobenzofuranone, 3-butyl-, 3-butylphthalide, butylphthalide, N-butylphthalide	chemical drug	61361	--	
2	albiflorin	Albiflorin; 39011-90-0; Alibiflorin; Albiflorin;Ambap39011-90-0	biologic drug	51346141		
3，5	alpha-tocopherol	2H-1-benzopyran-6-ol, 3,4-dihydro-2,5,7,8-tetramethyl-2-((4R,8R)-4,8,12-trimethyltridecyl)-, (2R)-, 59-02-9, (2R)-2,5,7,8-tetramethyl-2-[(4R,8R)-4,8,12-trimethyltridecyl]-3,4-dihydrochromen-6-ol, C29H50O2, D-alpha-tocopherol, D-α-tocopherol, α-tocopherol	chemical drug	14985	HMDB0001893	59-02-9
3，5	apigenin	4H-1-benzopyran-4-one, 5,7-dihydroxy-2-(4-hydroxyphenyl)-, 4H-1-benzopyran-4-one, 5,7-dihydroxy-2-(4-hydroxyphenyl)- (9CI), 5,7,4'-trihydroxyflavone, 5,7-dihydroxy-2-(4-hydroxyphenyl)chromen-4-one, 520-36-5, C15H10O5, flavone, 4',5,7-trihydroxy-	chemical - endogenous non-mammalian	5280443	HMDB0002124	520-36-5
5	astragalin	3,4',5,7-tetrahydroxyflavone-3-glucoside, 5,7-dihydroxy-2-(4-hydroxyp	chemical - endogenous non-mammal	5282102	--	480-10-4

		henyl)-3-[(2S,3R,4S,5S,6R)-3,4,5-trihydroxy-6-(hydroxymethyl)oxan-2-yl]oxychromen-4-one, 480-10-4, C21H20O11, kaempferol 3-O-beta-D-glucopyranoside, kaempferol 3-O-β-D-glucopyranoside, kaempferol-3-O-glucoside, trifolin	ian			
4	azelaic acid	1,7-heptanedicarboxylic acid, 1,9-nonanedioic acid, 123-99-9, 17265-13-3, 17356-30-8, 19619-43-3, 27825-99-6, 38900-29-7, 52457-54-2, azelaic acid, potassium salt, Azelex, C9H16O4, dilithium azelate, dipotassium azelate, disodium azelate, dodium hydrogen azelate, Emerox 1110, Emerox 1144, Emery's L-110, Finacea, Finevin, nonanedioic acid, Skinoren	chemical - endogenous mammalian	2266	HMDB0000784	27825-99-6
3	baicalin	7-D-glucuronic acid-5,6-dihydroxy-flavone, 21967-41-9, (2S,3S,4S,5R,6S)-6-(5,6-dihydroxy-4-oxo-2-phenylchromen-7-yl)oxy-3,4,5-trihydroxyoxane-2-carboxylic acid, baicalein 7-O-glucuronide, baicalein-7-D-glucuronide, beta-D-glucopyranosiduronic acid, 5,6-dihydroxy-4-oxo-2-phenyl-4H-1-benzopyran-7-yl, C21H18O11, β-D-glucopyranosiduronic acid, 5,6-dihydroxy-4-oxo-2-phenyl-4H-1-benzopyran-7-yl	chemical - endogenous non-mammalian	64982	HMDB0041832	21967-41-9
3,5	caffeic acid	2-propenoic acid, 3-(3,4-dihydroxyphenyl)-,	chemical drug	2518	HMDB0001964	331-39-5

		2-propenoic acid, 3-(3,4-dihydroxyphenyl)-(9CI), 3,4-dihydroxybenzeneacrylic acid, 3,4-dihydroxycinnamic acid, 3-(3,4-dihydroxyphenyl)prop-2-enoic acid, 4-(2'-carboxyvinyl)-1,2-dihydroxybenzene, 4-(2-carboxyethenyl)-1,2-dihydroxybenzene, 331-39-5, C9H8O4				
3	carnosol	2H-9,4a-(epoxymethano)phenanthren-12-one, 1,3,4,9,10,10a-hexahydro-5,6-dihydroxy-1,1-dimethyl-7-(1-methylethyl)-, (4aR-(4aalpha,9alpha,10abeta))-, 5957-80-2, 93241-27-1, C20H26O4	chemical - endogenous non-mammalian	442009	HMDB0002121	5957-80-2
5	carthamin yellow	Saffron powder	biologic drug			1401-20-3
4	carvacrol	2-hydroxy-p-cymene, 2-methyl-5-propan-2-ylphenol, 2-p-cymenol, 5-isopropyl-2-methylphenol, 499-75-2, antioxine, C10H14O, isopropyl-o-cresol, isothymol, karvakrol, o-thymol, p-cymen-2-ol	chemical - endogenous non-mammalian	10364	HMDB0035770	499-75-2
5, 3	chlorogenic acid	3-(3,4-dihydroxycinnamoyl)quinic acid, 3-caffeoylquinic acid, 327-97-9, (1S,3R,4R,5R)-3-[(E)-3-(3,4-dihydroxyphenyl)prop-2-enoyl]oxy-1,4,5-trihydroxycyclohexane-1-carboxylic acid, C16H18O9, chlorogenate, cyclohexanecarboxylic acid, 3-((3-(3,4-dihydroxyphenyl)-1-oxo-2-propenyl)oxy)-1,4,5-trihydroxy-,	chemical drug	1794427	HMDB0003164	327-97-9

		(1S-(1-alpha,3-beta,4-alpha,5-alpha))-, cyclohexanecarboxylic acid, 3-((3-(3,4-dihydroxyphenyl)-1-oxo-2-propenyl)oxy)-1,4,5-trihydroxy-, (1S-(1-α,3-β,4-α,5-α))-, trans-5-O-caffeoyl-D-quinate, trans-5-O-caffeoyl-D-quinic acid				
3	cryptotanshinone	35825-57-1, (1R)-1,6,6-trimethyl-2,7,8,9-tetrahydro-1H-naphtho[1,2-g][1]benzofuran-10,11-dione, C19H20O3, phenanthro(1,2-b)furan-10,11-dione, 1,2,6,7,8,9-hexahydro-1,6,6-trimethyl-, (R)-	chemical drug	160254	HMDB0035220	35825-57-1
3	danshensu		biologic drug	11600642		
1, 3, 4	ferulic acid	2-Propenoic acid, 3-(4-hydroxy-3-methoxyphenyl)-, 4-hydroxy-3-methoxycinnamic acid, 537-98-4, 1135-24-6, 24276-84-4, (E)-3-(4-hydroxy-3-methoxyphenyl)prop-2-enoic acid, C10H10O4, sodium ferulate	chemical - endogenous non-mammalian	445858	HMDB0000954	24276-84-4
2	gallic acid	3,4,5-trihydroxybenzoic acid, 149-91-7, benzoic acid, 3,4,5-trihydroxy-, C7H6O5, gallate	chemical - endogenous non-mammalian	370	HMDB0005807	149-91-7
5	hydroxysafflor yellow a		biologic drug	6443665		
5	kaempferol	3,5,7-trihydroxy-2-(4-hydroxyphenyl)chromen-4-one, 520-18-3, C15H10O6	chemical toxicant	5280863	HMDB0005801	520-18-3
5	lauric acid	1-undecanecarboxylic acid, 12:0 fatty acid, 143-07-7, 629-25-4, 10124-65-9, C12 fatty acid, C12:0, C12:0 free fatty acid, C12H24O2, dodecanoate, dodecanoic	chemical - endogenous mammalian	3893	HMDB0000638	10124-65-9

		acid, dodecylcarboxylate, laurate, n-dodecanoic acid, sodium dodecanoate, sodium laurate				
1, 5	linoleic acid	9,12-linoleic acid, 9,12-octadecadienoic acid (9Z,12Z)-, 9,12-octadecadienoic acid, (Z,Z)-, 9Z,12Z-linoleic acid, 60-33-3, 2197-37-7, (9Z,12Z)-octadeca-9,12-dienoic acid, alpha-linoleic acid, C18:2w6, C18H32O2, cis,cis-9,12-octadecadienoic acid, linoleate, linolelaidic acid, linolic acid, n-6 PUFA 18:2,6, octadecadienoic acid, α-linoleic acid	chemical - endogenous mammalian	5280450	HMDB0000673	60-33-3
3	lithospermic acid	28831-65-4; CHEMBL518243; UNII-100IP83JAC; Lithospermic-acid	chemical - endogenous non-mammalian	6441498		
3，5	luteolin	2-(3,4-dihydroxyphenyl)-5,7-dihydroxy-4-benzopyrone, 2-(3,4-dihydroxyphenyl)-5,7-dihydroxychromen-4-one, 3',4',5,7-tetrahydroxyflavone, 4H-1-benzopyran-4-one, 2-(3,4-dihydroxyphenyl)-5,7-dihydroxy- (9CI), 491-70-3, C15H10O6, cyanidenon 1470, digitoflavone, flacitran, luteolol	chemical - endogenous non-mammalian	5280445	HMDB0005800	491-70-3
3	luteoloside		biologic drug	5280637		
5	myricetin	3,5,7-trihydroxy-2-(3,4,5-trihydroxyphenyl)chromen-4-one, 529-44-2, C15H10O8	chemical - endogenous non-mammalian	5281672	HMDB0002755	529-44-2
3	oleanolic acid	3-beta-hydroxyolean-12-en-28-oic acid, 3-β-hydroxyolean-12-en-28-oic acid, 508-02-1, (4aS,6aR,6aS,6bR,8aR,10S,12aR,14bS)-10-hydroxy-2,2,6a	chemical - endogenous non-mammalian	10494	HMDB0002364	508-02-1

		,6b,9,9,12a-heptamethyl-1,3,4,5,6,6a,7,8,8a,10,11,12,13,14b-tetradecahydropicene-4a-carboxylic acid, astrantiagenin C, C30H48O3, caryophyllin, giganteumgenin C, olean-12-en-28-oic acid, 3-hydroxy-, (3beta)-, olean-12-en-28-oic acid, 3beta-hydroxy-, olean-12-en-28-oic acid, 3beta-hydroxy- (8CI), virgaureagenin B				
2	oxypaeoniflorin		biologic drug	21631105		
1，2，3，4，5	palmitic acid	16:0 fatty acid, 57-10-3, 143-20-4, c16 fatty acid, C16:0, C16:0 fatty acid, C16:0 free fatty acid, C16H32O2, cetylic acid, hexadecanoate, hexadecanoic acid, hexadecylic acid, n-hexadecanoic acid, palmitate, palmitate acid, sodium palmitate	chemical - endogenous mammalian	985	HMDB0000220	143-20-4
2	peoniflorin	5b-((benzoyloxy)methyl)tetrahydro-5-hydroxy-2-methyl-2,5-methano-1H-3,4-dioxacyclobuta(cd) pentalen-1a(2H)-yl-beta-D-glucopyranoside, 5b-((benzoyloxy)methyl)tetrahydro-5-hydroxy-2-methyl-2,5-methano-1H-3,4-dioxacyclobuta(cd) pentalen-1a(2H)-yl-β-D-glucopyranoside, 5b-((benzoyloxy)methyl)tetrahydro-5-hydroxy-2-methyl-2,5-methano-lH-3,4-dioxacyclobuta(cd)pentalen-1a(2H)-yl-beta-D-glucopyranoside, 5b-((benzoyloxy)methyl)tetrahydro-5-hydroxy-2-methyl-2,	chemical drug	5458396	--	23180-57-6

		5-methano-lH-3,4-dioxacyclobuta(cd)pentalen-1a(2H)-yl-β-D-glucopyranoside, 23180-57-6, beta-D-glucopyranoside, 5b-((benzoyloxy)methyl)tetrahydro-5-hydroxy-2-methyl-2,5-methano-1H-3,4-dioxacyclobuta(cd)pentalen-1a(2H)-yl, (1aR-(1a-alpha,2-beta,3a-alpha,5-alpha,5a-alpha,5b-alpha))-, $C_{23}H_{28}O_{11}$, paeonia moutan, paeoniflorin, paeony root, β-D-glucopyranoside, 5b-((benzoyloxy)methyl)tetrahydro-5-hydroxy-2-methyl-2,5-methano-1H-3,4-dioxacyclobuta(cd)pentalen-1a(2H)-yl, (1aR-(1a-α,2-β,3a-α,5-α,5a-α,5b-α))-				
5	quercetin	2-(3,4-dihydroxyphenyl)-3,5,7-trihydroxychromen-4-one, 3,3',4,5,7-pentahydroxyflavone, 3,5,7,3',4'-pentahydroxyflavone, 4H-1-benzopyran-4-one, 2-(3,4-dihydroxyphenyl)-3,5,7-trihydroxy-, 117-39-5, $C_{15}H_{10}O_7$, cyanidanol, flavin meletin, meletin, quercetin dihydrate, quercitin	chemical drug	5280343	HMDB0005794	117-39-5
3	rosmarinic acid	3-(3,4-dihydroxyphenyl)-2-[(E)-3-(3,4-dihydroxyphenyl)prop-2-enoyl]oxypropanoic acid, 537-15-5, benzenepropanoic acid, alpha-((3-(3,4-dihydroxyphenyl)-1-oxo-2-propenyl)oxy)-3,4-dihydroxy-, benzenepropanoic acid, α-((3-(3,4-dihydroxyphenyl)-1-oxo-2-propenyl)oxy)-3,4-dihydroxy-, $C_{18}H_{16}O_8$, rosmarinate	chemical - endogenous non-mammalian	5315615	--	537-15-5

3, 5	rutin	2-(3,4-dihydroxyphenyl)-5,7-dihydroxy-3-[(2S,3R,4S,5S,6R)-3,4,5-trihydroxy-6-[[(2R,3R,4R,5R,6S)-3,4,5-trihydroxy-6-methyloxan-2-yl]oxymethyl]oxan-2-yl]oxychromen-4-one, 3,3',4',5,5',7-hexahydroxyflavone (6-O-alpha-L-rhamnosyl-beta-D-glucoside), 3,3',4',5,5',7-hexahydroxyflavone (6-O-α-L-rhamnosyl-β-D-glucoside), 3,3',4',5,7-pentahydroxyflavone-3-rutinoside, 4H-1-benzopyran-4-one, 3-((6-O-(6-deoxy-alpha-L-mannopyranosyl)-beta-D-glucopyranosyl)oxy)-2-(3,4-dihydroxyphenyl)-5,7-dihydroxy-, 4H-1-benzopyran-4-one, 3-((6-O-(6-deoxy-α-L-mannopyranosyl)-β-D-glucopyranosyl)oxy)-2-(3,4-dihydroxyphenyl)-5,7-dihydroxy-, 153-18-4, C27H30O16, glucopyranoside, quercetin-3 6-O-alpha-L-rhamnopyranosyl-, beta-D, glucopyranoside, quercetin-3 6-O-α-L-rhamnopyranosyl-, β-D, phytomelin, quercetin-3-rutinoside, rutoside	chemical toxicant	5280805	HMDB0003249	153-18-4
5	safflor yellow A		biologic drug	71463725		
3	salvianolic acid A	SALVIANOLIC ACID; (R)-3-(3,4-Dihydroxyphenyl)-2-(((E)-3-(2-((E)-3,4-dihydroxystyryl)-3,4-dihydroxyphenyl)acryloyl)oxy)propanoic acid	biologic drug	5281793		96574-01-5
3	salvianolic		biologic drug	11629084		

	acid B					
2	sitosterol	3beta-sitosterol, 5779-62-4, (3S,8S,9S,10R,13R,14S,17R)-17-[(2R,5R)-5-ethyl-6-methylheptan-2-yl]-10,13-dimethyl-2,3,4,7,8,9,11,12,14,15,16,17-dodecahydro-1H-cyclopenta[a]phenanthren-3-ol, C29H50O	chemical - endogenous non-mammalian	222284	--	5779-62-4
2	syringin	118-34-3, (2R,3S,4S,5R,6S)-2-(hydroxymethyl)-6-[4-[(E)-3-hydroxyprop-1-enyl]-2,6-dimethoxyphenoxy]oxane-3,4,5-triol, C17H24O9, eleutheroside B, lilacin, methoxyconiferine, syringenin	chemical - endogenous non-mammalian	5316860	--	118-34-3
3	ursolic acid	77-52-1, (1S,2R,4aS,6aR,6aS,6bR,8aR,10S,12aR,14bS)-10-hydroxy-1,2,6a,6b,9,9,12a-heptamethyl-2,3,4,5,6,6a,7,8,8a,10,11,12,13,14b-tetradecahydro-1H-picene-4a-carboxylic acid, C30H48O3	chemical drug	64945	HMDB0002395	77-52-1

Note：1. *Chuanxiong Rhizoma* (Chuanxiong); 2. *Paeoniae Radix Rubra* (Chishao); 3. *Salviae miltiorrhizae* (Danshen); 4. *Angelicae sinensis Radix* (Danggui); 5. *Carthami Flos* (Honghua).
